# Laser Additively Manufactured High-Entropy Alloys via Laser Powder Bed Fusion and Laser-Directed Energy Deposition: Process–Structure–Property Relationships and Design Strategies

**DOI:** 10.3390/ma19153190

**Published:** 2026-07-26

**Authors:** Meng-Yun Lee, Hyoung Seop Kim, An-Chou Yeh

**Affiliations:** 1 Department of Materials Science and Engineering, National Tsing Hua University, 101, Sec. 2, Kuang-Fu Road, Hsinchu 300044, Taiwan; 2 Department of Materials Science and Engineering, Pohang University of Science and Technology (POSTECH), Pohang 37673, Republic of Korea; hskim@postech.ac.kr; 3 High-Entropy Materials Center, National Tsing Hua University, 101, Sec. 2, Kuang-Fu Road, Hsinchu 300044, Taiwan; 4 Graduate Institute of Ferrous & Eco Materials Technology, Pohang University of Science and Technology (POSTECH), Pohang 37673, Republic of Korea; 5 Institute of Space Engineering, National Tsing Hua University, 101, Sec. 2, Kuang-Fu Road, Hsinchu 300044, Taiwan

**Keywords:** high-entropy alloys, laser powder bed fusion, laser-directed energy deposition, printability, defect formation, residual stress, mechanical properties, CALPHAD

## Abstract

High-entropy alloys (HEAs) offer attractive combinations of mechanical performance, thermal stability, and compositional flexibility, making them promising candidates for advanced structural applications. Laser-based additive manufacturing, particularly laser powder bed fusion (LPBF) and laser-directed energy deposition (LDED), enables the fabrication of geometrically complex HEA components with non-equilibrium microstructures. However, the distinct thermal histories of LPBF and LDED, with typical cooling rates of approximately 10^5^–10^7^ K s^−1^ and 10^2^–10^4^ K s^−1^, respectively, strongly govern solidification behavior, elemental segregation, residual stress development, defect formation, and mechanical properties. Although previous reviews have discussed additively manufactured HEAs, an integrated framework linking composition design, printability, LPBF/LDED processing, microstructural evolution, post-processing, and industrial qualification remains limited. Therefore, this review establishes a unified composition–process–structure–property framework for laser additively manufactured HEAs. Fundamental HEA concepts, LPBF/LDED process characteristics, solidification behavior, phase formation, defect evolution, and mechanical performance from ambient to elevated temperatures are systematically discussed across representative FCC, refractory, and dual-phase HEA systems. This review emphasizes that printability should be considered during alloy design by correlating composition-dependent solidification characteristics, cracking susceptibility, phase stability, and defect formation with mechanical performance. Post-processing treatments are shown to modify residual stress, microsegregation, precipitation behavior, porosity, and deformation mechanisms, although their benefits must be balanced against thermal softening or brittle phase formation. Finally, CALPHAD, integrated computational materials engineering (ICME), machine learning (ML), and in situ monitoring are identified as promising tools for accelerating alloy and process optimization, while reproducible process windows, defect-control criteria, databases, and qualification protocols remain essential for industrial implementation.

## 1. Introduction

High-entropy alloys (HEAs) were first introduced in the early 2000s [1,2] and are typically defined as alloys containing five or more principal elements in near-equiatomic composition. Conventional alloys are usually based on one or two dominant elements, whereas HEAs consist of complex multicomponent compositions that have historically been associated with the so-called four core effects of high-entropy effect, severe lattice distortion, sluggish diffusion, and the cocktail effect [3,4]. With these characteristics, HEAs provide a favorable combination of mechanical properties and thermal stability [5,6,7], making HEAs promising candidates for demanding structural applications. Among the various HEA systems, 3D transition metal HEAs (3D-TM HEAs), such as CoCrFeNi-based alloys [8,9,10,11] and CoCrFeMnNi-based alloys [12,13,14,15], are widely investigated due to their excellent tensile performance associated with face-centered-cubic (FCC) structure and deformation mechanisms such as twinning-induced plasticity (TWIP) and transformation-induced plasticity (TRIP) [10,15]. Refractory HEAs (RHEAs) consist of high-melting-point elements, such as Nb, Mo, Ta, and W [16], and typically exhibit body-centered-cubic (BCC) structures. RHEAs are well-known for their superior high-temperature strength [17] and excellent high-temperature oxidation resistance [18] with limited room-temperature ductility [17].

However, conventional fabrication methods for HEAs typically require extensive thermomechanical processing, which can limit compositional exploration, restrict microstructural optimization, and constrain fabrication efficiency. In contrast, additive manufacturing (AM) offers a viable alternative by enabling the fabrication of complex, multi-component alloys with high spatial flexibility and accuracy. AM provides significant advantages, including geometric freedom, enhanced design flexibility, reduced material waste, and shortened processing routes. AM processes include binder-based AM, laser-based AM, and liquid-based AM. Within various AM processes, the laser-based AM processes [19], including laser powder bed fusion (LPBF) [20,21,22,23,24,25] and laser-directed energy deposition (LDED) [26,27,28,29,30], have attracted considerable attention because of their high-energy laser sources and precise control over processing parameters. The rapid cooling rate of up to 10^6^ K/s, steep thermal gradients, and high internal stress induced by laser-based processes can promote the hierarchically heterogeneous microstructures within HEAs, including melt-pool boundaries, columnar grains, fine grains, and cellular substructures decorated with high-density dislocations and microsegregation. Localized heating and rapid solidification can also influence the phase formation, texture, and defect evolution, resulting in a distinct mechanical performance of laser-based AM-processed HEAs.

Despite these inherent advantages, several key challenges remain in the laser-based manufacturing of HEAs and can generally be categorized into process-induced defects and composition-dependent defects. Process-induced defects primarily originate from the complex thermal cycles and melt-pool dynamics associated with laser-based AM. During LPBF and LDED, the mismatch in the coefficient of thermal expansion between the deposited HEAs and the substrate, combined with repeated layer-by-layer heating and cooling, can generate severe residual thermal stresses [29,31]. These residual stresses not only cause distortion in the final parts but also constrain the formability of large-scale components because the accumulated thermal strain increases with deposition thickness. Furthermore, inappropriate processing parameters, such as insufficient or excessive laser energy input and unsuitable scanning speeds, can destabilize the melt pool and promote the formation of defects, including lack-of-fusion (LoF) porosity, keyhole pores, and gas porosity. Such defects significantly deteriorate densification behavior and ultimately compromise the mechanical performance of the fabricated HEAs. In contrast, composition-dependent defects are strongly associated with the intrinsic complexity of multicomponent HEAs and their non-equilibrium solidification behavior during laser processing. The extreme cooling rates inherent to LPBF and LDED inevitably promote elemental microsegregation because of the substantial disparities in atomic radii and diffusion rates among the multiple principal elements [26]. The segregation of alloying elements toward submicron cellular boundaries facilitates the precipitation of undesirable brittle phases, severely compromises local plasticity, and increases susceptibility to crack initiation. Moreover, the evaporation of low-melting-point elements under excessive local heat accumulation can induce unintended compositional deviations, further affecting phase stability, segregation behavior, and cracking susceptibility.

Several previous review articles have provided important foundations for understanding HEAs and their additive manufacturing. Prior reviews have critically assessed the founding principles of HEAs, including the core effects, phase stability, and alloy selection strategies [32]. Other studies have summarized the relationships among synthesis routes, microstructural evolution, and mechanical properties of HEAs produced by casting, powder metallurgy, and additive manufacturing [33]. Early reviews on AM-processed HEAs mainly focused on the development of bulk HEAs, with emphasis on microstructural evolution, mechanical properties, post-heat treatment effects, and general processing challenges [34]. More recent reviews further expanded the discussion to various AM technologies for HEAs, including powder bed fusion, directed energy deposition, binder jetting, and material extrusion [19]. In parallel, broader reviews on AM metals have highlighted the roles of non-equilibrium solidification, hierarchical microstructures, processing defects, and micromechanical deformation mechanisms in determining mechanical performance [35]. Although these studies have substantially advanced the understanding of HEAs and AM metals, they generally emphasize either HEA fundamentals, general synthesis routes, broad AM process classifications, or mechanical behavior across different metallic materials. A focused review that directly compares LPBF- and LDED-processed metallic HEAs within a unified composition–process–structure–property framework remains limited. In particular, the relationships among composition-dependent printability, process-specific defect formation, AM-induced hierarchical microstructures, post-processing-induced retention or degradation of strengthening mechanisms, and mechanical performance have not yet been fully integrated.

To address these gaps, the present review focuses primarily on metallic HEAs fabricated by LPBF and LDED, which are representative laser-based AM techniques characterized by rapid solidification and steep thermal gradients. Other laser processing technologies, including laser cladding, laser remelting, and hybrid manufacturing approaches, are beyond the primary scope of this review and are discussed only when they provide relevant insights into phenomena associated with LPBF and LDED processing. This review systematically correlates alloy composition, laser processing conditions, solidification behavior, microstructural evolution, defect formation, post-processing responses, and mechanical properties. Particular emphasis is placed on identifying HEA systems with favorable printability for LPBF and LDED, clarifying the dominant process- and composition-related defects associated with each process, such as LoF porosity, keyhole porosity, residual stress-induced cracking, solidification cracking, and microsegregation, and assessing how post-processing treatments, including stress relief, homogenization, aging, hot isostatic pressing (HIP), and cryogenic treatment, preserve, modify, or reduce AM-induced hierarchical microstructures and strengthening mechanisms. Furthermore, the roles of CALPHAD- and artificial intelligence-assisted composition optimization are critically discussed to highlight their potential for accelerating the development of printable laser-based AM HEAs with tailored properties. Finally, the remaining scientific and technological challenges, together with future research directions toward industrial implementation, are discussed.

## 2. Fundamentals of HEAs

HEAs are generally defined as multicomponent alloys containing several principal elements, typically with each element present between 5 and 35 at%. The behavior of HEAs has historically been discussed in terms of four so-called core effects [3,4]; however, recent studies emphasize that these effects should be treated as heuristic concepts rather than universal mechanisms. Therefore, the following discussion first summarizes these effects in their conventional thermodynamic and metallurgical contexts, and then relates them to the non-equilibrium solidification characteristics of laser-based AM. For laser-based AM HEAs, their relevance must be reconsidered in the context of non-equilibrium solidification, microsegregation, and process-induced defect structures. The high-entropy effect has often been invoked as a thermodynamic factor contributing to solid-solution stabilization, although phase formation in HEAs is also strongly governed by enthalpic interactions, atomic-size mismatch, valence electron concentration, and processing history. Thermodynamically, a system reaches equilibrium by minimizing its Gibbs free energy at a given temperature and pressure. Accordingly, the Gibbs free energy of mixing ($∆G_{mix}$) can be expressed as $∆G_{mix}=∆H_{mix}-T∆S_{mix}$, where $∆H_{mix}$ is the mixing enthalpy, T is the temperature, and $∆S_{mix}$ is the mixing entropy. The incorporation of multiple principal elements increases the configurational entropy of mixing, thereby reducing the Gibbs free energy of the system. This results in promoting the formation of simple solid-solution phases and suppressing the formation of brittle intermetallic compounds, often resulting in fewer phases than those predicted by the Gibbs phase rule and thus enhancing high-temperature phase stability. However, it is difficult to explore the stable phase regions of the HEA systems with multiple elements by trial-and-error experimental observation. In this context, the CALPHAD approach has been employed to predict phase stability and phase diagrams of HEAs by integrating thermodynamic databases with computational modeling. This approach minimizes deleterious phase formation and reduces alloy development costs, although its predictive accuracy remains dependent on the reliability of available thermodynamic databases. HEAs are also characterized by severe lattice distortion arising from the presence of constituent elements with varying atomic radii and bonding energies, forcing the atoms to deviate from ideal lattice sites and, as a result, accumulate lattice strain. This distortion impedes dislocation movement and contributes to pronounced solid-solution strengthening, thereby enhancing hardness and yield strength. In addition, the distorted lattice introduces spatial variations in potential energy, forming localized energy barriers that increase the activation energy for atomic diffusion. This so-called sluggish diffusion effect reduces atomic mobility, thereby delaying phase transformations, suppressing precipitate coarsening, and improving high-temperature stability and creep resistance. The cocktail effect describes the synergistic interactions among multiple elements, which enable HEAs to exhibit properties that exceed those predicted by the rule of mixtures. Through compositional design, HEAs can be tailored to achieve optimized combinations of mechanical strength, creep resistance, and oxidation resistance, making them promising candidates for advanced structural applications. In laser-based AM, these core effects remain relevant but must be interpreted in the context of non-equilibrium solidification. Unlike equilibrium or near-equilibrium processing, LPBF and LDED involve rapid cooling, steep thermal gradients, and repeated thermal cycling, which impose strong kinetic constraints on phase formation [19,29,31]. Therefore, the high-entropy effect contributes to solid-solution stabilization, but the final phase constitution is also affected by rapid-cooling-induced phase retention and limited atomic redistribution. Severe lattice distortion is also modified by AM-induced elemental microsegregation, cellular dislocation substructures, and residual microstresses, making the local lattice strain spatially heterogeneous. Similarly, the suppression of diffusion-controlled transformations in as-built laser-based AM HEAs may result from both intrinsic sluggish diffusion and the limited atomic redistribution during rapid solidification. Accordingly, the four core effects should be regarded as composition-related fundamentals that interact with laser-based AM thermal history, microsegregation, residual stress, and process-induced defects.

The deformation mechanisms of FCC HEAs are strongly associated with stacking fault energy (SFE), which influences competition among twinning-induced plasticity (TWIP) [10] and transformation-induced plasticity (TRIP) [15]. In conventional FCC alloys and several representative FCC HEAs, an intermediate SFE range of approximately 20–40 mJ/m^2^ is often considered favorable for planar glide and the formation of deformation twins during plastic deformation. These deformation twin boundaries can act as potent obstacles to dislocation motion, resulting in dislocation accumulation and enhanced work-hardening capacity. Consequently, the TWIP mechanism can endow FCC HEAs with a favorable synergy of strength and ductility. When the SFE is reduced below approximately 20 mJ/m^2^, strain-induced phase transformation may be promoted, and the dominant deformation mode can shift toward the TRIP effect. In FCC HEAs, this transformation generally involves martensitic transformation from the parent FCC phase to a hexagonal close-packed (HCP) phase. The phase transformation process not only dissipates deformation energy but also introduces a high density of newly generated FCC/HCP-phase boundaries. These interfaces further enhance resistance to dislocation glide, resulting in a stable strain-hardening rate that effectively overcomes the traditional strength–ductility trade-off. However, these numerical SFE ranges should be regarded as empirical guidelines rather than universal boundaries for all FCC HEAs because the critical SFE for activating TWIP or TRIP depends strongly on alloy composition, temperature, chemical short-range order, grain size, strain rate, and processing history. In contrast, the fundamental deformation mechanisms of BCC HEAs are strongly influenced by lattice distortion and the dynamics of screw dislocations [16,36,37]. Unlike the closely packed FCC lattice, the more open BCC structure accommodates greater atomic displacement, thereby inducing more pronounced lattice distortion in the presence of significant atomic-size mismatch. Severe lattice distortion promotes kink-pair nucleation on screw dislocations and can generate cross-kinked structures within the BCC lattice, which present higher energy barriers for dislocation glide and result in immense slip resistance. During plastic deformation, the heavily constrained dislocation mobility leads to extensive dislocation multiplication and elevated internal stresses. Coupled with the intrinsically limited dislocation mobility of BCC structures, this restricted movement manifests as straight, parallel slip bands and high-density dislocation networks. Ultimately, excessive lattice distortion and severe pinning of dislocation motion can trigger rapid saturation of dislocation density. This inevitably promotes strain localization, causing a sharp deterioration in the work-hardening rate and a concomitant loss of ductility [38].

Furthermore, the deformation mechanisms of HEAs are highly temperature-dependent and are frequently interpreted through the homologous temperature ($T/T_{m}$) [39,40,41,42]. Homologous temperature provides a useful framework for comparing deformation behavior across alloy systems with different melting points; however, the boundaries between different deformation regimes should be regarded as approximate guidelines rather than universal thresholds. These regimes can shift depending on alloy chemistry, crystal structure, phase stability, SFE, grain size, strain rate, and processing history. Variations in homologous temperature generally dictate the dominant thermal activation processes and subsequent defect evolution. At cryogenic temperatures, corresponding to extremely low $T/T_{m}$, thermal activation energy is severely restricted, which constrains conventional dislocation glide. Concurrently, the SFE of many FCC HEAs decreases as the temperature decreases. Consequently, deformation in low-SFE FCC HEAs may be increasingly assisted by TWIP and TRIP, imparting a high work-hardening rate and good strength–ductility synergy. At ambient-temperature regimes, approximately $0.1\;<\;T/T_{m}\;<\;0.3$, increased thermal activation enhances dislocation mobility, resulting in a combined deformation mode in which dislocation slip can operate together with TWIP and/or TRIP effects. As the temperature increases to the intermediate-to-elevated regime, approximately $0.3\;<\;T/T_{m}\;<\;0.5$, sufficient thermal activation can facilitate dislocation climb and cross-slip. The enhanced dislocation mobility may suppress TWIP and TRIP effects, leading to more homogeneous stress and strain distributions. However, this generally occurs at the expense of work-hardening capacity and yield strength due to thermal softening. Finally, in high-temperature regimes, often corresponding to $T/T_{m}\;>\;0.5$, elevated thermal energy can promote dislocation annihilation, grain-boundary migration, dynamic recrystallization, and diffusion-assisted deformation. Depending on the strain rate and stress state, the deformation behavior may gradually transition into creep-related mechanisms, which are dominated by viscous glide at low strain rates and dislocation climb at high strain rates. Meanwhile, extreme high-temperature exposure can exacerbate elemental segregation or grain-boundary weakening in some HEAs, thereby increasing susceptibility to intergranular cracking.

## 3. Laser-Based Additive Manufacturing Techniques

### 3.1. Laser Powder Bed Fusion (LPBF)

**Figure 1:**
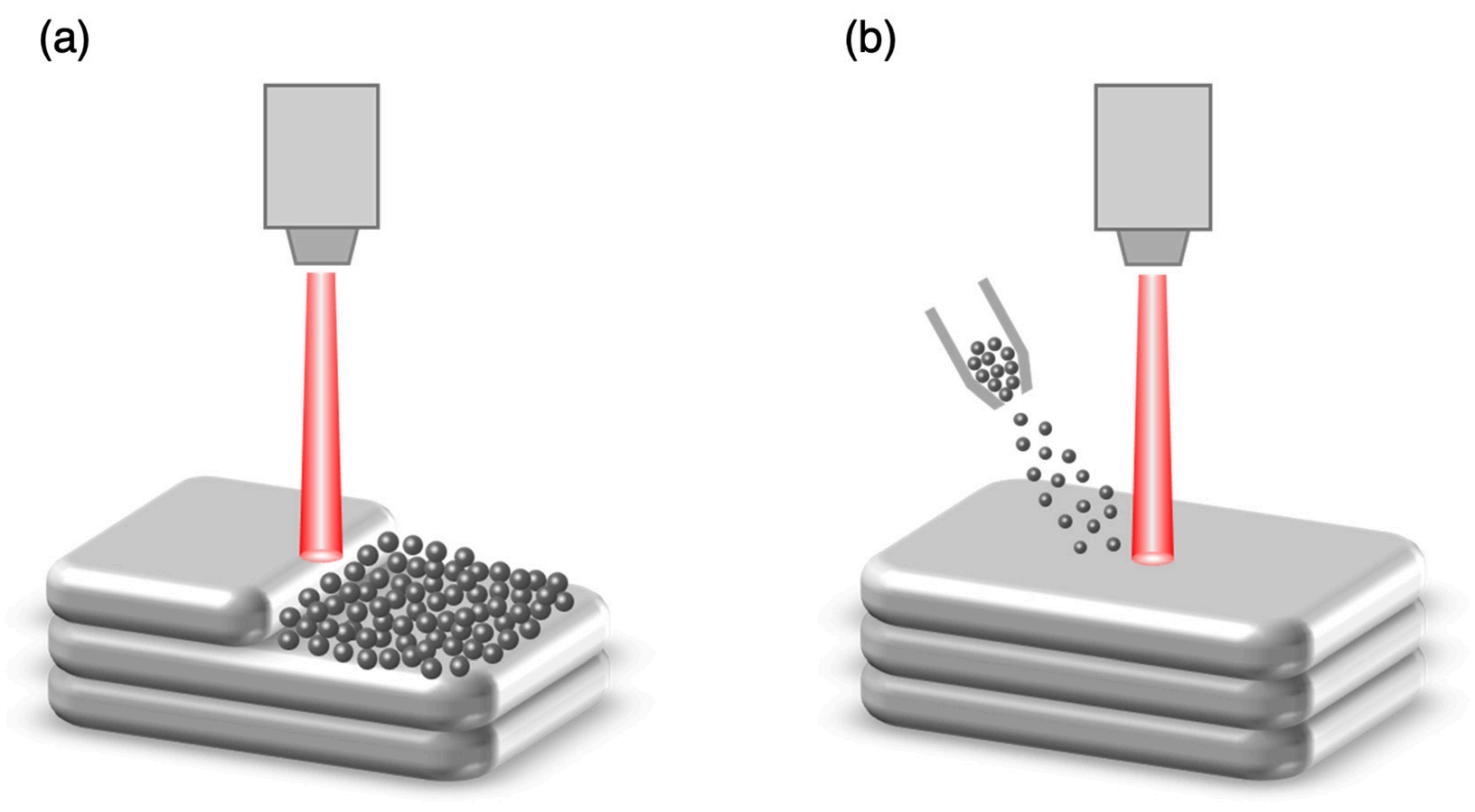
The schematic of the (**a**) LPBF and (**b**) LDED processes.

LPBF is one of the most widely used AM techniques, in which a high-energy laser selectively melts powder layers to fabricate complex metallic components [20,21,22,23,24,25]. The schematic of the LPBF process is shown in Figure 1a. To mitigate the internal stresses induced by extreme thermal gradients, the substrate is typically pre-heated, which reduces cracking susceptibility during the LPBF process. Furthermore, the processing chamber is rigorously purged and filled with an inert argon atmosphere to control the oxygen concentration, thereby preventing detrimental oxidation in the high-temperature environment. For HEA fabrication, feedstock selection represents an additional processing issue because the compositional uniformity of multicomponent alloys is strongly affected by the initial powder state. Elemental blended powders offer high compositional flexibility and lower costs, making them useful for rapid alloy screening. However, differences in the melting point, density, particle size, diffusivity, and laser absorptivity among the constituent powders may cause nonuniform melting, incomplete atomic diffusion, local chemical segregation, and residual unmelted particles during rapid melting and solidification. In contrast, pre-alloyed powders prepared by gas atomization or plasma spheroidization generally provide more uniform composition within each particle and are therefore preferred for producing high-density HEA parts with reduced segregation. Nevertheless, their composition is fixed during powder production, which limits rapid compositional tuning [25]. Powder handling influences LPBF stability. Variations in powder flowability, apparent density, and tap density directly affect the quality and packing uniformity of the powder bed. Irregular particles can introduce local variations in powder-bed density, leading to spatial fluctuations in laser absorption and melt-pool stability. These variations may promote stochastic pore formation, LoF defects, and local compositional fluctuations. Advanced powder conditioning methods, such as radio-frequency plasma spheroidization, can improve particle sphericity, flowability, and tap density, thereby enhancing powder-bed uniformity and process stability [25]. During the LPBF process, manufacturing parameters play a crucial role in governing the densification behavior and the subsequent microstructural evolution of the as-built parts, and the laser parameters are summarized in Table 1. The critical processing parameters primarily encompass laser power, scanning speed, hatch distance, powder layer thickness, laser spot size, and the interlayer rotation angle. In particular, the volumetric laser energy density (VED) significantly influences the melting characteristics of the powder bed and the final microstructural features. The VED (E), defined as the laser energy input per unit volume of the material, can be quantitatively expressed by the empirical equation E=P/(v×h×d), where P represents the laser power, v is the scanning speed, h is the hatch distance, and d is the powder layer thickness. For HEAs containing volatile elements, such as Mn- or Al-containing alloys, excessive energy input can cause selective evaporation because these elements have relatively low boiling points and high vapor pressures [43,44]. Such evaporation may shift the final alloy composition away from the designed HEA composition and disturb phase stability. In addition, metal vaporization can generate unstable recoil pressure and spatter, which further increases the risk of keyhole porosity. For refractory HEAs, oxygen control is another critical concern. Elements such as W, Mo, Nb, Ta, Ti, and Zr exhibit strong affinity for oxygen, and oxygen adsorbed on powder surfaces or residual oxygen in the chamber can be absorbed by the high-temperature melt pool. Subsequent grain-boundary oxygen segregation or nanoscale oxide formation may weaken grain-boundary cohesion and promote intergranular cracking, which is particularly detrimental to the already limited room-temperature ductility of RHEAs [25].

### 3.2. Laser-Directed Energy Deposition (LDED)

LDED is a laser-based AM process in which powder or wire feedstock is delivered into a melt pool generated by a focused laser beam [26,27,28,29,30], as shown in Figure 1b, and the laser parameters are summarized in Table 1. Depending on the feedstock delivery mode, LDED can be generally classified into powder-fed and wire-fed configurations. The powder-fed approach, which is the most widely adopted configuration, typically utilizes a coaxial nozzle to deliver powders under an inert protective atmosphere. This feeding strategy provides a strong in situ alloying capability because the feeding rates of multiple elemental or pre-alloyed powders can be independently adjusted. Therefore, powder-fed LDED is particularly attractive for exploring complex HEA compositions and fabricating functionally graded materials with spatially tailored compositions.

However, powder-fed LDED also presents several limitations. Differences in particle size, morphology, density, flowability, laser absorptivity, and melting temperature among constituent powders may lead to differential melting, incomplete mixing, compositional inconsistencies, microsegregation, and local chemical fluctuations. In HEA fabrication, in situ alloying is often performed by mechanically blending pre-alloyed powders such as CoCrFeNi with additional elemental powders, such as Ti, Al, or refractory elements [28,29]. Although this strategy expands the accessible compositional space, maintaining feedstock uniformity during blending, transportation, and powder delivery remains challenging. If powder segregation occurs because of differences in particle density or size, the actual composition delivered into the melt pool may fluctuate significantly. This feedstock nonuniformity can induce macrosegregation, retain unmelted refractory particles, or cause abnormal enrichment of hard and brittle phases, thereby increasing the risk of defect formation and cracking. In addition, powder catchment efficiency is sensitive to the powder feed rate, carrier gas flow, nozzle geometry, standoff distance, and track overlap ratio, which may further influence deposition stability, dilution, porosity, and material utilization. Carrier gas flow must be carefully controlled because unstable powder delivery can change the local powder concentration entering the melt pool and lead to bead-shape fluctuations. These issues are more pronounced in multicomponent HEAs than in conventional alloys because small deviations in local composition may alter phase stability, segregation behavior, and cracking susceptibility.

Conversely, the wire-fed method utilizes solid metal wires as feedstock, usually in the form of pre-alloyed wires. Compared with powder-fed LDED, wire-fed LDED generally offers higher material utilization efficiency because feedstock scattering and powder overspray can be largely avoided. It is also advantageous for large-scale repair and bulk deposition when the target alloy composition is already fixed. Nevertheless, its compositional flexibility is relatively limited because the available alloy compositions are restricted by commercially available or custom-made wires. This limitation is particularly important for HEAs, where rapid compositional screening and local compositional tuning are often required. Therefore, powder-fed LDED is more suitable for in situ alloy design and functionally graded HEA fabrication, whereas wire-fed LDED is more attractive for high-efficiency deposition of predetermined HEA compositions. In terms of industrial applications, LDED is mainly used for targeted repair, coating, and the fabrication of large or near-net-shape components.

### 3.3. Process Comparison

**Figure 2:**
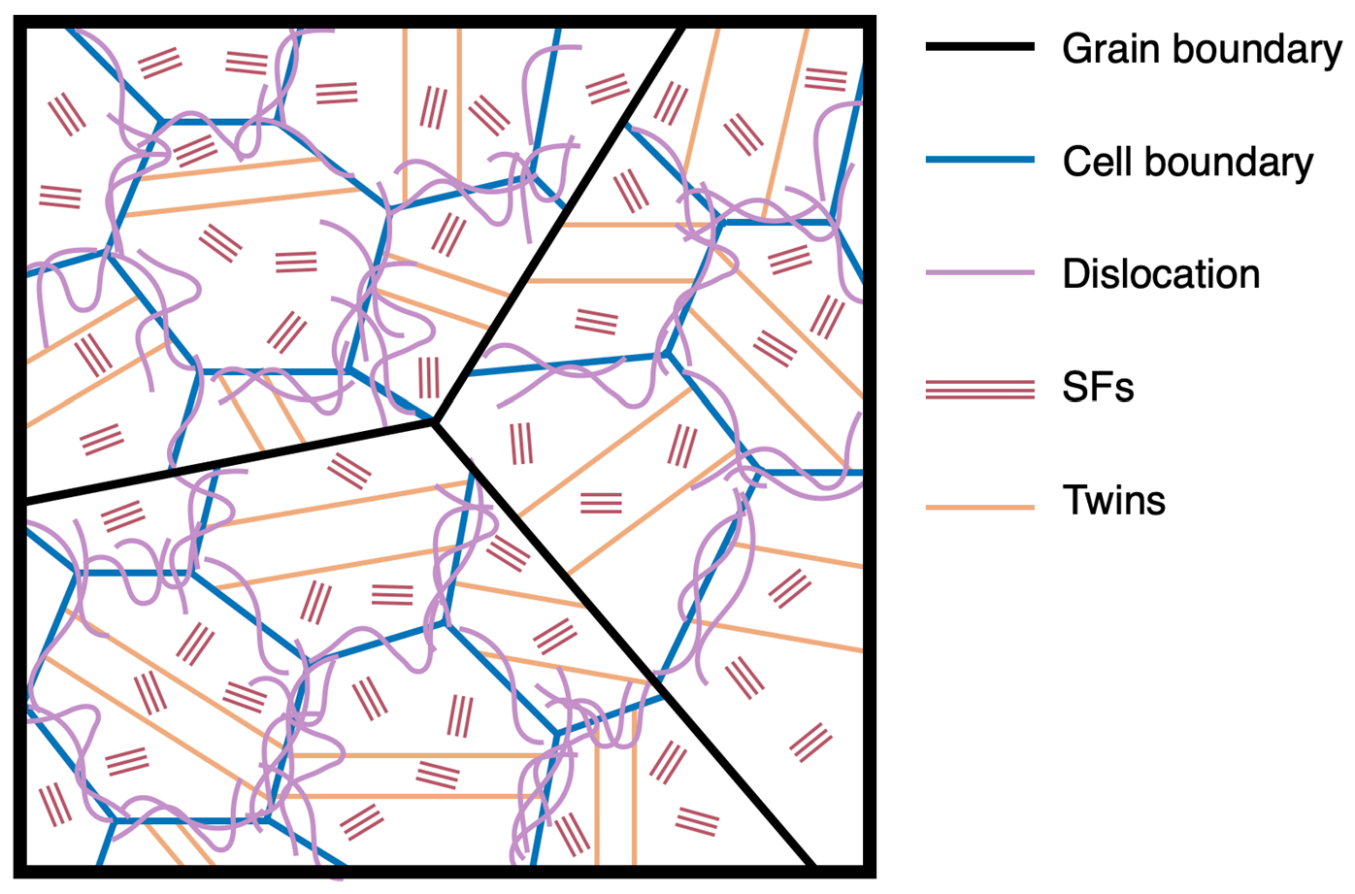
The schematic of HEAs fabricated by laser-based AM processes, consisting of dislocation cell structures and SFs.

Both LPBF and LDED involve localized melting and rapid solidification, but their thermal histories differ markedly because of differences in laser power, laser spot size, scan speed, layer thickness, and feedstock delivery mode. These differences strongly affect melt-pool geometry, solidification behavior, defect formation, microstructural anisotropy, elemental segregation, as well as the final mechanical properties. The comparison of thermal history during LPBF, LDED, and conventional processing is summarized in Table 2. Figure 2 schematically illustrates the characteristic microstructural features of HEAs fabricated by laser-based AM processes. During these processes, the high-energy laser input generates extreme thermal gradients and severe internal strains. The synergistic effect of large thermal gradients and rapid solidification within the molten pool facilitates the epitaxial growth of columnar grains along the heat dissipation direction.

The solidification microstructure in laser-based AM can be interpreted using two key thermal parameters: the temperature gradient (G) and the solidification rate (R). The ratio G/R governs the solidification mode and the transition of microstructures from planar growth to cellular, columnar dendritic, and equiaxed morphologies. A high G/R ratio generally favors directional epitaxial growth, whereas a lower G/R ratio promotes constitutional supercooling and facilitates the columnar-to-equiaxed transition (CET). In contrast, the product G × R represents the local cooling rate, which controls the degree of undercooling, nucleation rate, and characteristic microstructural length scale [45]. Therefore, higher G × R values usually lead to finer grains, reduced dendrite/cell spacing, and stronger grain-refinement effects [27]. Within a single melt pool, G and R vary significantly with spatial position. At the melt-pool bottom and fusion boundary, heat is mainly extracted into the previously solidified substrate or deposited layer. As a result, G is high while R is relatively low, giving rise to a high G/R ratio. This condition suppresses equiaxed nucleation and promotes the epitaxial growth of columnar grains along the direction of maximum heat dissipation [46,47]. As the solidification front moves toward the melt-pool center and top surface, heat extraction becomes less directional, thermal accumulation increases, G decreases, and R increases. The resulting lower G/R ratio enhances constitutional supercooling and may trigger CET, leading to fine equiaxed grains or disordered cellular/dendritic structures near the melt-pool top [47]. This spatial variation explains why laser-based AM HEAs often exhibit mixed columnar–equiaxed microstructures and location-dependent textures.

Compared with LDED, LPBF typically uses a lower laser power, smaller laser spot size, thinner powder layers, and a much higher scan speed. These conditions generate a small melt pool with a high thermal gradient of approximately 10^4^–10^7^ K m^−1^ and an ultrahigh cooling rate of 10^5^–10^7^ K s^−1^. The rapid solidification strongly restricts long-range atomic diffusion, thereby reducing coarse segregation and suppressing the formation of some brittle secondary phases [48]. The high cooling rate also promotes refined grains and a submicron cellular dislocation structure, which contributes to a high yield strength and tensile strength. However, the concentrated energy input in LPBF can also induce keyhole porosity, spatter, and strong residual thermal stresses. In high-strength HEAs and RHEAs, these stresses increase the risk of thermal cracking [49]. Moreover, strong directional heat extraction often promotes columnar grain growth and crystallographic texture along the building direction, resulting in mechanical anisotropy [50]. In contrast, LDED generally uses higher laser power, a larger laser spot size, thicker deposition layers, and a lower scan speed. These conditions produce a larger melt pool, stronger thermal accumulation, and a lower cooling rate, typically in the range of 10^2^–10^4^ K s^−1^. The slower solidification provides more time for solute redistribution, which can intensify elemental segregation and promote the precipitation or coarsening of secondary phases [51]. Meanwhile, because powder-fed LDED relies on synchronous powder delivery, defects are strongly affected by feedstock uniformity and powder catchment stability. Unmelted particles, especially high-melting-point refractory elements such as Mo and W, can remain in the deposited layer under insufficient energy input [52]. These particles, together with macrosegregation and gas entrapment in the large melt pool, may serve as crack initiation sites.

LPBF and LDED also differ in their anisotropy and heterogeneity. In LPBF, anisotropy is mainly associated with directional epitaxial growth, strong crystallographic texture, and columnar grains aligned along the building direction. Therefore, tensile properties measured parallel and perpendicular to the building direction can differ significantly [50]. In LDED, the larger melt pool and repeated thermal cycling generate stronger location-dependent heterogeneity along the build height. The bottom region generally experiences stronger substrate cooling and tends to contain coarse columnar grains, whereas the middle and top regions are more affected by heat accumulation, remelting, and reduced G/R values. This can result in gradual changes in grain morphology, phase fraction, segregation degree, and mechanical response from the bottom to the top of the deposited part [53].

Overall, LPBF is more suitable for fabricating small and geometrically complex HEA components with high resolution and high strength, owing to its fine microstructure, high cooling rate, and strong microstructural refinement. Nevertheless, its application is limited by the build size, residual stress, keyhole/LoF defects, and anisotropy. LDED is more suitable for large-scale manufacturing, repair, coating, and functionally graded HEAs because of its high deposition rate and flexible feedstock delivery. However, its lower cooling rate and powder-fed nature increase the risk of coarse grains, segregation, unmelted refractory particles, macrosegregation, and location-dependent properties. Therefore, selecting LPBF or LDED for HEA fabrication requires a combined consideration of thermal parameters, melt-pool geometry, feedstock stability, defect tolerance, microstructural anisotropy, and the required balance between strength and ductility.

**Table 1:** Laser parameters of LPBF and LDED processes.

Parameter	LPBF	LDED	Influence on HEAs	Ref.
Laser power	50–400 W [31,54,55,56]	600–4500 W; up to ~12 kW for some RHEAs [52,57,58]	Melt-pool size, porosity, dilution	[31,52,54,55,56,57,58]
Scan speed	600–1200 mm s^−1^ [55,56,59,60]	4–20 mm s^−1^ [19,52,61]	Cooling rate, LoF/keyhole transition	[19,52,55,56,59,60,61]
Layer thickness	20–50 μm [20,55,62,63]	0.2–0.8 mm [52,57]	Resolution, thermal accumulation	[20,52,55,57,62,63]
Spot size	50–100 μm [20,46,55]	1–5 mm [26,52,64]	Build rate, grain size	[20,26,46,52,55,64]
Hatch spacing/track overlap	40–100 μm [55,59,65,66]	0.5–2.5 mm or 30–50% overlap ratio [64,67,68,69]	Controls inter-track bonding, porosity, residual stress distribution, and microstructural uniformity	[55,59,64,65,66,67,68,69]
Powder feed rate	Not applicable	3–34 g min^−1^	Affects deposition rate, powder catchment efficiency, dilution, and compositional stability	[52,58,61,68]
Shield/carrier gas	Inert chamber gas, typically Ar, N_2_ [20,70,71]	Shield gas, usually Ar; carrier gas, usually Ar or He [58,61,72,73]	Influences oxidation, powder delivery stability, spatter behavior, and oxygen pickup in oxygen-sensitive RHEAs	[20,58,61,70,71,72,73]
Standoff distance	Not applicable	Typically 10–20 mm	Affects powder stream focusing, catchment efficiency, bead geometry, dilution, and surface quality	[57,67]
Feedstock	Powder bed [31,55]	powder/wire feeding [58,67]	Composition flexibility	[31,55,58,67]
Typical application	Complex small parts [54,74]	repair/large parts/FGM [45,64,75]	Process selection	[45,54,64,74,75]

**Table 2:** Comparison of thermal history during various processes.

	LPBF Process	LDED Process	Conventional Process	Ref.
Cooling rate (K s^−1^)	Ultrafast (10^5^~10^7^)	Fast (10^2^~10^4^)	Slow (10^−1^~10^1^)	[76]
Thermal gradient (K m^−1^)	Extremely high (10^6^~10^7^)	High (10^4^~10^6^)	Low (10^2^~10^4^)	[76]
Melt pool size	Microscale	Meso to Macroscale	Macroscale	[31]

## 4. Process–Structure Relationships

### 4.1. Solidification Behavior

LPBF involves highly non-equilibrium solidification under steep thermal gradients and high cooling rates, typically in the range of 10^5^–10^7^ K/s [76]. Owing to the small laser spot size, thin powder layer, and fast scanning speed, the melt pool is relatively small and solidifies rapidly. These conditions promote a diffusion-limited solidification regime, in which long-range atomic diffusion and extensive elemental partitioning are strongly restricted. As a result, LPBF generally produces finer microstructures than conventional casting or thermomechanical processing [65]. Rapid solidification facilitates hierarchical microstructures, typically characterized by submicron cellular structures with high-density dislocation entanglement along cell boundaries [21,22,71]. Although solute redistribution is limited by the short solidification time, differences in atomic radius, melting point, and diffusion coefficient among constituent elements can still promote microsegregation at interdendritic or cellular boundaries. These microsegregated regions may act as nucleation sites for secondary phases, such as L2_1_ phases [21]. In addition, repeated remelting between adjacent tracks and successive layers is essential for metallurgical bonding in LPBF [62]. The relatively shallow remelting depth and localized dilution help maintain fine cellular or columnar substructures [64], while excessive or insufficient overlap can, respectively, promote heat accumulation or LoF defects.

In contrast, LDED generally operates with a larger laser spot size, higher laser power, thicker deposited layers, and slower scanning speeds, resulting in lower cooling rates, typically in the range of 10^2^–10^4^ K/s [76]. Compared with LPBF, the larger melt pool and longer thermal exposure in LDED provide more time for solute redistribution, making the solidification behavior closer to diffusion-mediated solidification. Consequently, LDED-fabricated HEAs are more prone to pronounced microsegregation, coarser dendritic or cellular structures, and the precipitation or coarsening of secondary phases. Because of the thicker layer thickness, the temperature gradient and solidification rate can vary substantially within a single deposited layer, leading to location-dependent microstructures [29]. The bottom region of the melt pool usually experiences high G and low R conditions and therefore tends to exhibit planar or epitaxial growth from the underlying layer. The middle region often develops columnar grains along the preferred heat-flow direction, whereas the upper region may contain cellular, dendritic, or partially equiaxed grains due to reduced thermal gradients and altered G/R conditions.

Thermal accumulation is also more pronounced in LDED than in LPBF. In LPBF, repeated reheating occurs during layer-by-layer fabrication, but the small melt-pool volume and relatively rapid heat dissipation through the powder bed and substrate tend to confine heat accumulation to local regions. In LDED, the larger heat input and lower heat dissipation efficiency cause the local temperature gradient and cooling rate to decrease progressively with increasing build height. This thermal accumulation promotes coarser microstructures and contributes to the strong location dependence of grain morphology, phase distribution, and mechanical properties [53]. Furthermore, although both LPBF and LDED involve epitaxial growth from the remelted substrate or previously deposited layer, the tendency for columnar-to-equiaxed transition differs. LPBF commonly favors continuous epitaxial columnar growth along the maximum heat-flow direction because of its steep thermal gradients [64]. In LDED, epitaxial growth is also observed near the melt-pool bottom, but the lower G/R ratio and stronger thermal accumulation in the upper melt-pool region can promote columnar-to-equiaxed transition, resulting in a higher fraction of equiaxed grains or mixed columnar/equiaxed grain structures [64].

Another important difference lies in the remelting depth and dilution. In LPBF, remelting is generally confined to a shallow region of the previous layer, which helps preserve fine-scale solidification structures while ensuring interlayer bonding [64]. In LDED, the deeper melt pool and continuous material feeding lead to stronger dilution between the substrate and the deposited HEA layer. This dilution modifies local composition, thermal flow, and solidification behavior near the interface. For example, in LDED-processed AlCoCrFeNi HEA, Al and Ni segregation in dendritic regions promotes B2 phase formation, whereas Fe and Cr enrichment in interdendritic regions favors BCC phase formation [27]. Therefore, compared with LPBF, LDED solidification is more strongly affected by thermal accumulation, deeper remelting, dilution, and location-dependent solute redistribution, which together produce more pronounced gradient microstructures.

### 4.2. Phase Formation in Laser-Processed HEAs

The extreme thermal gradients and rapid cooling rates inherent to laser-based additive manufacturing strongly influence phase formation and microstructural metastability in HEAs. Although the high configurational entropy can reduce the Gibbs free energy of multicomponent solid solutions, phase selection is not governed by entropy alone. Instead, it is jointly controlled by valence electron concentration (VEC), atomic-size mismatch, mixing enthalpy, electronegativity difference, and the non-equilibrium thermal history imposed by LPBF or LDED. Therefore, empirical phase-selection descriptors and CALPHAD-based phase-field predictions are useful for rationalizing the formation of FCC, BCC, and dual-phase HEAs during laser-based AM.

Among these descriptors, VEC is commonly used to estimate the preferred crystal structure of solid-solution HEAs. In general, alloys with a VEC ≥ 8.0 tend to form FCC solid solutions, whereas alloys with a VEC ≤ 6.8 preferentially form BCC solid solutions. When the VEC lies between approximately 6.8 and 8.0, FCC and BCC phases are more likely to coexist, leading to dual-phase or eutectic microstructures [48,77,78]. However, VEC alone cannot capture the formation of intermetallic compounds or topologically close-packed phases. Atomic-size mismatch (δ) and mixing enthalpy ($\Delta H_{mix}$) provide additional information on solid-solution stability [79]. Random solid solutions are usually favored when δ is relatively small and $\Delta H_{mix}$ is moderately negative or near zero, whereas large atomic-size mismatch and strongly negative $\Delta H_{mix}$ promote chemical ordering, lattice instability, and intermetallic phase formation. The thermodynamic parameter Ω, defined by the balance between mixing entropy and mixing enthalpy, is also frequently used to assess whether a solid-solution phase is stable [47]. In addition, electronegativity difference is important for predicting brittle secondary phases. For example, a large atomic-size mismatch combined with a large Allen electronegativity difference can increase the tendency for Laves phase formation [47].

CALPHAD calculations further provide a thermodynamic basis for phase prediction beyond empirical descriptors. By combining thermodynamic databases with equilibrium phase diagrams or Scheil solidification simulations, CALPHAD can estimate primary solidification phases, solidification ranges, solute segregation paths, and the possible precipitation of secondary phases such as *γ*′, Laves, or B2 phases [79]. This is particularly important for laser-based AM because rapid solidification and repeated thermal cycling can retain metastable phases or shift precipitation behavior away from equilibrium predictions. CALPHAD can also be used to evaluate phase stability at service temperatures and to estimate the driving force for transformations such as FCC-to-HCP transformation, which is relevant to the design of TWIP- or TRIP-assisted HEAs [15]. Nevertheless, these descriptors and calculations should be considered as complementary tools because phase formation in laser-based AM HEAs is also affected by the local cooling rate, melt-pool geometry, elemental evaporation, dilution, and thermal accumulation.

Based on their dominant crystal structures, laser-based AM HEAs can generally be classified into FCC, BCC, and dual-phase HEAs. FCC HEAs are commonly composed of 3D transition metals, such as Co, Cr, Fe, Mn, and Ni, with the equiatomic CoCrFeMnNi HEA, also known as the Cantor alloy, being the most widely studied composition [2]. These alloys usually possess high VEC values and relatively moderate atomic-size mismatch, which favor FCC solid-solution formation. The close-packed FCC lattice provides multiple independent slip systems, giving these alloys good ductility and fracture toughness [22]. When the Cantor alloy is fabricated by LPBF and LDED, both techniques can produce a single-phase FCC structure dominated by columnar grains. However, their different thermal histories lead to clear microstructural differences. The average grain size of LDED-fabricated samples is approximately twice that of LPBF-fabricated counterparts. Furthermore, process-specific defects are prevalent: spherical Mn oxides tend to preferentially form at the top region of LDED samples due to in situ oxidation, whereas the LPBF process is inherently more susceptible to LoF and balling defects [62].

BCC HEAs are typically composed of refractory elements, including Nb, Mo, Ta, W, V, Zr, and Hf. These elements generally lower the VEC and favor BCC solid-solution formation. However, the large atomic-size mismatch and strong chemical interactions in many refractory HEAs can also promote local lattice distortion, elemental segregation, or brittle secondary phases. While BCC refractory HEAs exhibit high yield strength and good high-temperature stability [52], their room-temperature ductility is often limited, making them susceptible to brittle fracture [25]. Therefore, for AM-processed RHEAs, phase-selection descriptors should be evaluated together with oxygen sensitivity, solidification range, and CALPHAD-predicted phase stability to reduce cracking and undesirable phase formation.

Dual-phase HEAs are usually obtained when the VEC falls between the FCC- and BCC-favored regimes or when BCC-stabilizing elements are added to an FCC matrix. Elements such as Al, Ti, W, and Mo can shift the phase stability toward BCC or ordered B2 phases, resulting in FCC + BCC/B2 dual-phase microstructures. A representative example is the AlCoCrFeNi_2.1_ eutectic HEA (EHEA) [44]. In this type of alloy, the FCC phase provides plastic deformability, whereas the BCC/B2 phase contributes to strengthening. The resulting properties depend strongly on phase fraction, lamellar spacing, coherency, and the thermal history imposed by LPBF or LDED. Therefore, the design of dual-phase HEAs for laser-based AM should combine empirical descriptors, such as the VEC, δ, $\Delta H_{mix}$, and electronegativity difference, with CALPHAD-predicted phase fields to balance solidification behavior, phase stability, and mechanical performance.

### 4.3. Defects and Their Origins

During laser-based additive manufacturing processes, inappropriate processing parameters can severely promote defect formation, which significantly compromises the densification behavior of the fabricated parts and subsequently deteriorates their overall mechanical properties. The following section systematically introduces various types of process-induced defects and elucidates their underlying formation mechanisms.

#### 4.3.1. Pores

LoF defects typically emerge under conditions of low VED or high scanning speeds [44,80]. During the additive manufacturing process, such insufficient energy input fails to completely melt the precursor powders or adequately penetrate the underlying deposited layers, resulting in poor metallurgical bonding between adjacent melt pools. Morphologically, LoF pores generally exhibit highly irregular shapes and frequently encapsulate unmelted powder particles. These defects severely disrupt microstructural continuity and act as potent stress concentration sites, thereby substantially deteriorating the mechanical performance of the fabricated components. Conversely, excessive laser energy input or exceptionally low scanning speeds facilitate the formation of keyhole pores [44,80]. Intense heat input triggers severe metal vaporization, generating an immense recoil pressure that deeply depresses the liquid metal and renders the bottom of the melt pool highly unstable. The repeated formation and collapse of this deep, narrow melt pool trap the metal vapor within the deposition layer before it can escape, ultimately forming spherical keyhole defects. Furthermore, metallurgical or gas pores can arise when residual gases within the powder feedstock or the inert shielding atmosphere from the build chamber are entrapped in the melt pool by intense Marangoni convection [26,80]. Particularly under relatively low laser energy inputs, the increased viscosity of the molten liquid, coupled with the intrinsically high solidification rates of the AM process, hinders the upward escape of gas bubbles. Consequently, these entrapped gases are frozen into the matrix, resulting in the formation of fine, spherical metallurgical pores.

In addition to laser energy input, shielding gas composition and oxygen control play critical roles in pore formation and related surface defects. Residual oxygen and moisture in the build chamber or powder feedstock can alter the surface-tension gradient of the molten pool, thereby modifying Marangoni convection and destabilizing the melt track [31,80]. Such melt-pool instability may promote balling and poor surface quality. Excessive oxygen exposure can also induce brittle oxide inclusions [31], particularly in HEAs containing oxygen-sensitive elements such as Al, Ti, Cr, Mn, or refractory elements [52]. These oxide particles tend to accumulate near melt-pool boundaries, cell boundaries, or grain boundaries, where they may act as local stress concentrators and crack-initiation sites. The choice of shielding gas composition can further influence melt-pool dynamics and microstructural evolution. High-purity Ar is commonly used because of its relatively inert behavior during laser-based AM. In contrast, when N_2_ is used as the shielding gas, nitrogen may dissociate or dissolve into the molten pool under high-energy laser irradiation and act as an interstitial solute. For refractory HEAs, oxygen or nitrogen may provide interstitial strengthening at low concentrations, but excessive interstitial uptake can deteriorate ductility and increase embrittlement susceptibility [52]. Moreover, shielding gas flow dynamics strongly influence the removal of metal vapor, fumes, and spatter from the laser interaction zone [80]. If the gas flow is insufficient or unstable, large spatter particles may redeposit onto the powder bed and remain partially unmelted during subsequent scanning, thereby increasing the probability of LoF pores and surface irregularities.

The distinct origins of these pore types indicate that pore mitigation should be tailored to whether the defect is caused by insufficient melting, excessive vaporization, gas entrapment, or oxygen-assisted melt-pool instability. LoF defects can be reduced by optimizing the VED through proper combinations of laser power, scanning speed, hatch distance, and layer thickness, thereby improving melt-pool penetration and overlap between adjacent scan tracks. Unmelted particles and spatter-induced defects can be further alleviated by increasing laser energy input within an appropriate processing window, applying laser remelting, and optimizing shielding gas flow to remove fumes and spatter from the powder bed. In contrast, keyhole porosity should be mitigated by avoiding excessive laser energy input or overly low scanning speeds, which helps suppress severe vaporization, recoil-pressure instability, and deep keyhole collapse. Metallurgical gas pores and oxide-related defects can be minimized by strict oxygen and moisture control in the build chamber, the use of high-purity inert shielding gas, and powder sieving or recycling control to remove oxidized, irregular, or satellite powders. In addition to in-process control, HIP can be employed as an effective post-build strategy to close internal pores and improve densification, as discussed in detail in Section 7.

#### 4.3.2. Cracking

Solidification cracking predominantly occurs in the central region of the melt pool during the terminal stage of solidification, specifically within the mushy zone where the solid fraction exceeds 90% [49,80]. The fundamental physical mechanism governing this phenomenon is well elucidated by the Rappaz–Drezet–Gremaud (RDG) model [81]. During the rapid cooling process, the rapid growth of dendrites drives the segregation of low-melting-point elements into the interdendritic regions, leading to the formation of continuous liquid films [82]. As adjacent dendrite arms impinge and coalesce, the residual liquid becomes isolated. Consequently, solidification shrinkage and thermally induced tensile strains cause a significant pressure drop within these localized regions. If the viscosity of the residual liquid is excessively high or the interdendritic channels are too narrow, adequate liquid backfilling is severely impeded. This feeding inadequacy inevitably results in the nucleation of shrinkage microvoids (pores). Once the accumulated residual thermal stress exceeds the local yield strength of the material, these pre-existing microvoids are mechanically torn apart, thereby initiating solidification cracks. Morphologically, such cracks typically exhibit a long and straight profile, frequently coupled with distinct dendritic features on the internal crack surfaces. Furthermore, solidification cracks preferentially propagate along high-angle grain boundaries (HAGBs). This susceptibility is attributed to the elevated grain boundary energy associated with high misorientations, which thermodynamically promotes the instability of the interdendritic liquid films, rendering these boundaries highly vulnerable to rupture.

As a critical process-induced defect, liquation cracking typically initiates within the heat-affected zone and is significantly exacerbated by the rapid heating and cooling cycles inherent to the additive manufacturing process [80,82]. The formation of liquation cracks is primarily governed by two distinct mechanisms. First, constitutional liquation occurs when solute elements are highly enriched at the matrix–precipitate interfaces. During the rapid heating stage, as the local temperature and solute concentration reach the eutectic reaction threshold, metastable liquid films form at these interfaces. These interconnected liquid films subsequently provide a continuous pathway for crack propagation throughout the heat-affected zone. Second, pre-existing low-melting-point secondary phases can be directly melted during the intense high-temperature thermal cycles, thereby generating additional liquid films. Morphologically, in contrast to the relatively straight profiles of solidification cracks, liquation cracks characteristically exhibit a more tortuous trajectory, frequently accompanied by the presence of re-solidified phases within the crack interior.

To clarify terminology overlap, cracking modes in laser-based AM HEAs can be distinguished according to the cracking temperature, the presence of liquid films, and fracture morphology. Hot cracking is an umbrella term that includes solidification cracking and liquation cracking [83]; both occur at high temperatures near the solidus and are associated with residual or locally re-melted liquid films. Strain-age cracking is also a solid-state cracking mode, but it is specifically related to rapid precipitation hardening during post-build thermal cycling or heat treatment, where matrix hardening limits stress relaxation [84]. This mechanism is further discussed as a printability criterion in Section 5.1. By contrast, cold cracking occurs after complete solidification at lower temperatures or near room temperature and is mainly driven by the interaction between high tensile residual stresses and limited local ductility [85]. Intergranular cracking should be regarded as a fracture morphology rather than a distinct cracking mechanism. Although hot cracks are often intergranular because liquid films preferentially form along grain boundaries, intergranular fracture in RHEAs can also occur in the solid state due to intrinsic brittleness and oxygen or interstitial segregation-induced grain-boundary embrittlement [25,86]. Therefore, intergranular cracking in RHEAs should not be automatically assigned to hot cracking unless liquid-film traces or other high-temperature cracking evidence is observed.

For the hot-cracking modes discussed above, cracking mitigation should simultaneously consider melt-pool stability, thermal-stress reduction, and the suppression of continuous low-melting-point liquid films. For solidification cracking, optimizing the VED is essential to maintain sufficient melt-pool penetration and stable track overlap while avoiding excessive overheating. Substrate preheating and optimized scan strategies can further reduce thermal gradients and residual tensile stresses accumulated during rapid solidification. For liquation cracking, the key mitigation strategy is to suppress the formation of low-melting liquid films. From the perspective of alloy design, elements or compositional combinations that promote low-melting eutectic constituents should be minimized to reduce the susceptibility to constitutional liquation. Segregation engineering is also critical because reducing solute enrichment at matrix/precipitate interfaces can decrease the tendency for local liquid-film formation during rapid reheating. From the processing perspective, optimized VED can prevent excessive heat accumulation, while substrate preheating and scan rotation can reduce thermal gradients during repeated laser thermal cycles. In contrast, strain-age cracking should be mitigated by controlling precipitation kinetics, residual stress, and post-build thermal exposure, and is further discussed as a printability criterion in Section 5.1.

#### 4.3.3. Residual Stress

**Figure 3:**
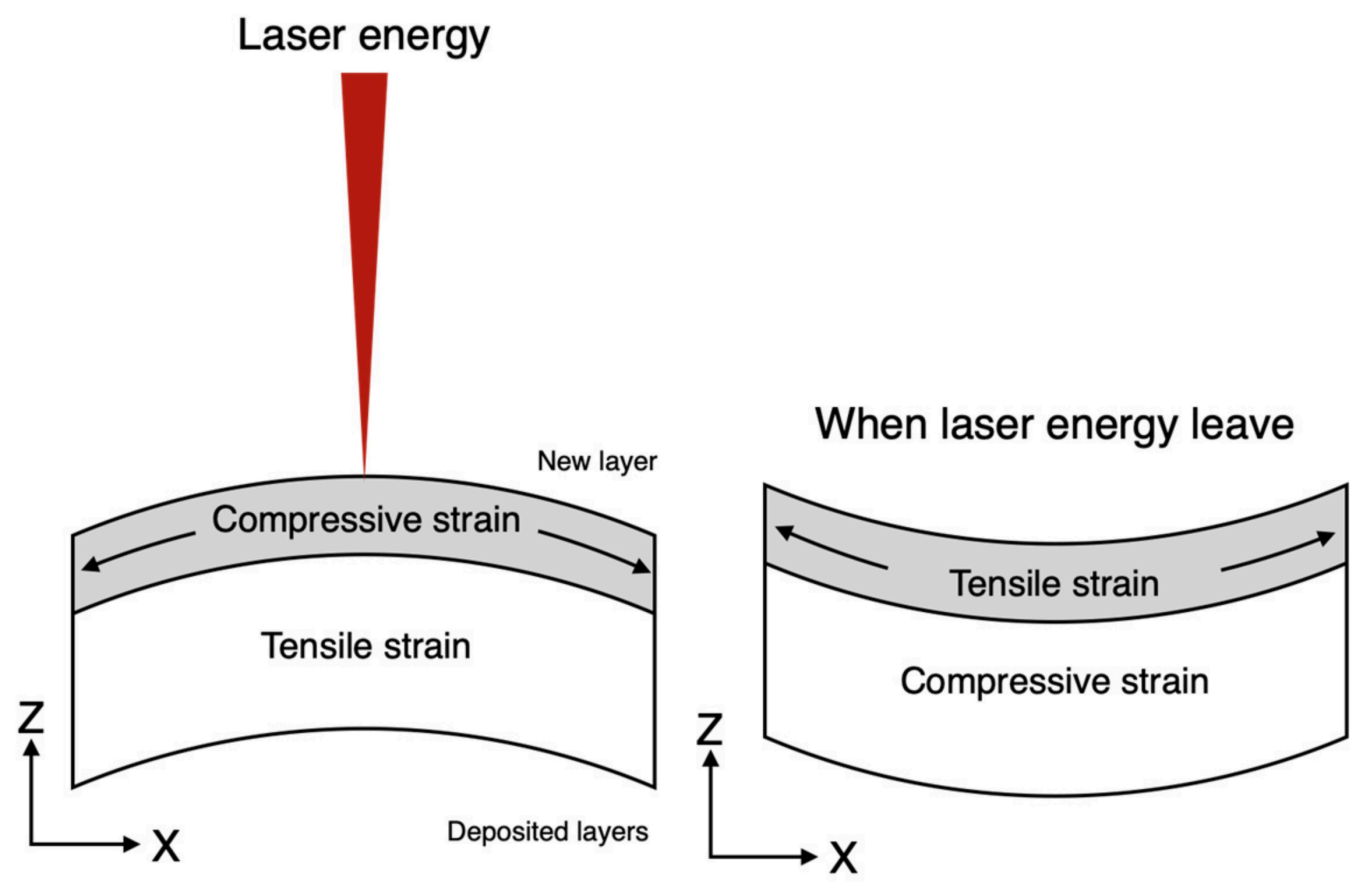
Residual stress induced by thermal-gradient mechanism.

In laser-based additive manufacturing, residual stresses inherently arise from complex thermomechanical interactions, predominantly manifesting as thermal and structural stresses [31]. The extreme thermal gradients and rapid cooling rates inherent to AM processes drive a temperature-gradient mechanism [87], as illustrated in Figure 3. During laser irradiation, the rapid thermal expansion of the top layer is strictly constrained by the underlying, cooler consolidated material, thereby generating localized compressive strains and stresses. Upon the departure of the heat source, the subsequent rapid cooling and shrinkage of this plastically compressed zone exert strong tensile forces on the surrounding matrix, ultimately leaving severe tensile residual thermal stresses within the as-built components. Furthermore, specific alloy systems undergo solid-state phase transformations during the rapid solidification process. The volumetric expansion associated with these phase changes, when constrained by the surrounding matrix, exacerbates the accumulation of structural residual stresses [29,31]. Beyond macroscopic stresses, the unique hierarchically heterogeneous microstructures characteristic of AM metals also induces localized microstresses [35]. The intergranular microstresses develop across adjacent grains to maintain self-equilibrium during load partitioning between plastically stiffer and softer grains. Concurrently, the intragranular microstresses can be induced due to severe local lattice distortions caused by high-density dislocation cell structures, chemical microsegregation, or nanoscale precipitates. Crucially, in the context of process-induced defects, these multiscale residual stresses act as the primary mechanical driving force, violently tearing pre-existing voids or vulnerable grain boundaries to initiate and propagate cracks.

Therefore, accurate characterization and prediction of residual stress are essential for evaluating the structural integrity of laser-based AM HEAs [88,89]. X-ray diffraction (XRD) is a widely used non-destructive technique that estimates residual stress from diffraction peak shifts caused by elastic lattice-strain variations. However, owing to its limited penetration depth, XRD is mainly suitable for near-surface residual stress measurements. In contrast, neutron diffraction provides a much greater penetration capability and can be used to determine internal residual stress distributions in bulk AM components, although its application is limited by the availability of neutron sources and relatively long measurement times. Mechanical relaxation methods, including the contour method and hole-drilling method, are also commonly employed. The contour method reconstructs through-thickness residual stress distributions from the deformation profile generated after sectioning a stressed component, while the hole-drilling method evaluates near-surface residual stress from local strain relaxation around a drilled hole. Digital image correlation (DIC), as a non-contact full-field deformation measurement technique, can be combined with hole drilling or sectioning methods to capture displacement and strain fields during stress release. In addition, finite-element simulation serves as an important complementary approach for predicting thermal stress evolution during laser-based AM and reconstructing residual stress fields from experimentally measured strain or deformation data.

Residual stress can be mitigated through both in-process thermal management and post-build heat treatments. During laser-based AM, substrate preheating is an effective strategy to reduce the temperature gradient between the molten pool and the underlying consolidated material, thereby decreasing tensile residual stress accumulation. Scan rotation and optimized scan-path design can also redistribute heat input and reduce localized stress concentrations. After fabrication, low-to-medium-temperature stress-relief heat treatment can relax residual stresses while partially preserving AM-induced cellular structures and dislocation networks, as discussed in Section 7.

#### 4.3.4. Interaction Between Defects and Deformation Mechanisms

Beyond their direct role as crack-initiation sites, process-induced defects can also interact with deformation mechanisms. LoF voids, keyhole pores, unmelted particles, and microcracks generate strong local stress concentrations, which may trigger the early activation of stacking faults (SFs), deformation twins, or strain-induced phase transformations around defect-affected regions. In low SFE FCC HEAs, such TWIP- or TRIP-assisted deformation can provide local plastic accommodation by relaxing strain incompatibility and delaying microcrack initiation. During crack propagation, TRIP-induced volume expansion may introduce local compressive residual stresses near the crack tip, promoting transformation-induced crack closure and reducing the effective crack-driving force. Meanwhile, high-density twin boundaries generated by the TWIP effect can redistribute localized strain, deflect crack paths, and increase the energy required for crack extension. Nevertheless, these beneficial mechanisms are highly sensitive to defect size, defect morphology, alloy composition, SFE, and phase stability. Severe LoF defects, keyhole porosity, or continuous cracks may still dominate failure before TWIP/TRIP-assisted work hardening can be fully activated.

### 4.4. Microstructural Anisotropy

The extreme thermal kinetics inherent to additive manufacturing processes dictate not only the formation of metallurgical defects but also the profound evolution of grain morphology and crystallographic texture. Specifically, the development of columnar grains and pronounced crystallographic textures fundamentally governs the mechanical anisotropy of AM-fabricated HEAs. Driven by the immense spatial temperature gradients, grains undergo preferential epitaxial growth at the solid–liquid interface, predominantly aligning with the direction of maximum heat dissipation, which typically parallels the building direction. This uninterrupted grain growth traversing multiple melt pools and deposited layers ultimately yields the coarse columnar grain architectures characteristic of AM metals [66,90]. Furthermore, crystallographic texture evolution is essentially dictated by competitive growth mechanisms, which are driven by both crystallographic thermodynamics and the complex geometric profiles of the melt pools. In FCC HEAs, the <001> crystallographic direction is typically the preferred solidification direction and tends to align with the thermal gradient during epitaxial growth. During rapid solidification, favorably oriented <001> grains that align with the local thermal gradient exhibit a superior growth velocity, effectively outcompeting misaligned grains, thereby culminating in a sharply defined crystallographic texture [59,66].

Build orientation further plays a critical role in determining the anisotropic mechanical properties of laser-based AM HEAs. Because the loading direction can be parallel, perpendicular, or inclined relative to the building direction, columnar grains, melt-pool boundaries, and layer interfaces, mechanical properties obtained from different orientations cannot be directly compared without specifying the specimen orientation. In tensile deformation, the anisotropy in yield strength is mainly governed by the effective grain size along the loading direction, crystallographic texture, Taylor factor, and heterogeneous deformation-induced hardening. For example, in LPBF-fabricated (FeCoNi)_86_Al_7_Ti_7_ HEA, the specimen loaded at 45° relative to the building direction exhibited the highest yield strength, which was attributed to its smaller effective grain size and higher Taylor factor [46]. In (CoCrFeMnNi)_99_C_1_ HEA, the tensile strength along the scanning direction was higher than those along other directions because the alternating fine- and coarse-grained layers promoted heterogeneous deformation-induced hardening through the accumulation of geometrically necessary dislocations and back stresses [66]. In addition to strength, room-temperature ductility can also show strong orientation dependence. For instance, the fracture elongation of the 0° specimen in (FeCoNi)_86_Al_7_Ti_7_ HEA reached 31.5%, whereas that of the 90° specimen decreased to 11.2%, demonstrating that build orientation can strongly affect ductility [46]. This orientation-dependent ductility is closely associated with fracture-path selection. When the tensile loading direction is perpendicular to the building direction, cracks tend to propagate across columnar grains, cellular substructures, and melt-pool boundaries. In this case, high-angle grain boundaries and heterogeneous microstructural interfaces can promote crack deflection, resulting in a tortuous mixed intergranular–transgranular fracture path and ductile dimpled fracture surfaces. Such crack-path tortuosity increases the energy required for crack extension and contributes to improved fracture resistance. In contrast, when the loading direction is parallel to the building direction, melt-pool boundaries and layer interfaces may become favorably oriented for decohesion under the applied tensile stress. Since these regions often contain residual thermal stresses, microsegregation, pores, or LoF defects, cracks can propagate rapidly along continuous melt-pool boundaries, leading to melt-pool-boundary decohesion, delamination-like fracture, and reduced tensile ductility.

The influence of build orientation also extends to fatigue behavior, but its effect should be distinguished between crack initiation and crack propagation stages [91]. During crack initiation, the anisotropic morphology of process-induced defects plays a dominant role. LoF defects are often flattened and distributed parallel to the deposited layers. When the loading direction is parallel to the building direction, these planar defects can present a larger projected area normal to the applied stress, thereby generating severe local stress concentrations and shortening fatigue crack-initiation life. In contrast, when the loading direction is perpendicular to the building direction, the projected defect area is reduced, leading to a lower stress concentration and a longer crack-initiation life. Therefore, the fatigue crack-initiation resistance generally follows the trend of 0° > 45° > 90°. During crack propagation, however, the anisotropic columnar grain structure becomes more important. For 90° specimens, crack propagation may be forced to cut across vertically aligned columnar grains and high-angle grain boundaries, resulting in pronounced crack deflection, crack branching, and a more tortuous crack path. This increases the energy required for crack extension and can reduce the fatigue crack-growth rate. In contrast, in 0° specimens, cracks may propagate more easily along columnar grain boundaries or layer-related interfaces, resulting in lower crack-growth resistance. Accordingly, the fatigue crack-propagation resistance can follow an opposite trend of 90° > 45° > 0°. Owing to these competing orientation-dependent mechanisms, the overall fatigue life is stress-level dependent. In the low-stress or high-cycle fatigue regime, fatigue life is mainly governed by crack initiation, and the larger projected defect area in the 90° orientation can lead to inferior fatigue performance. In the high-stress or low-cycle fatigue regime, cracks initiate rapidly, and the total fatigue life is affected by both initiation and propagation. Under such conditions, the 45° orientation may provide a more favorable balance between the moderate defect projected area and enhanced crack deflection, thereby yielding superior overall fatigue resistance. Therefore, the build orientation and loading direction should be explicitly reported when comparing tensile, fracture, and fatigue properties of laser-based AM HEAs since orientation-dependent microstructures and defect distributions can lead to substantially different mechanical responses.

To integrate the process–structure relationships discussed in Section 4.1, Section 4.2, Section 4.3 and Section 4.4, Table 3 summarizes the correlations among representative processing parameters, thermal conditions, microstructural outcomes, and defect formation in laser-based AM HEAs. This summary highlights that process parameters should not be considered independently, because energy input, spatial overlap, scan strategy, and thermal management collectively determine melt-pool stability, cooling behavior, residual stress, defect population, and mechanical anisotropy.

**Table 3:** Correlation between process parameters, microstructural outcomes, and typical defects or benefits in laser-based AM HEAs.

Process Parameter	Microstructural Outcome	Typical Defects/Benefits	Ref.
Low VED	Incomplete melting of powder and underlying layers; limited melt-pool fluidity	Irregular LoF defects, unmelted particles, reduced density	[73]
High VED	Enlarged melt pool; possible evaporation of low-boiling-point elements such as Mn or Al; local compositional deviation	Keyhole porosity, spatter, rough surface, thermal distortion, increased residual stress, higher cracking tendency	[73]
Large hatch spacing	Discontinuous tracks and poor inter-track bonding	Inter-track LoF pores, cracks, reduced density	[73]
Small hatch spacing	Excessive melt-pool overlap and local heat accumulation	Residual stress accumulation, rough surface, distortion	[73]
Thick layer thickness	Incomplete interlayer bonding; insufficient remelting of prior layer	Large interlayer LoF defects and unmelted powder	[92]
Thin layer thickness	Nearly fully dense structure and good surface quality	Significantly reduced production efficiency	[92]
Scan rotation strategy	Interrupts continuous epitaxial columnar growth; weakens strong crystallographic texture	Reduces texture-driven anisotropy	[52]
Preheating	Lower residual thermal stress; possible grain coarsening at excessive preheating temperature	Suppresses hot cracking and distortion; excessive preheating may promote grain growth or undesired phase precipitation	[93]
Remelting	Re-melts unmelted refractory particles; improves elemental homogenization; may partially relax stress through in situ thermal cycling	Reduces LoF defects, unmelted particles, and microsegregation; excessive remelting may increase heat accumulation	[52]
Short interlayer dwell time	Possible in situ aging or precipitation	Heat accumulation, residual stress redistribution, possible phase coarsening	[61]

### 4.5. Microstructure Comparison Between HEAs and Conventional Alloys Processed by Laser-Based AM

Conventional alloys processed by laser-based additive manufacturing often exhibit microstructural limitations associated with rapid non-equilibrium solidification. For example, Ni-based superalloys such as IN718, Hastelloy X, and Haynes 230 generally contain highly complex alloy chemistries with multiple secondary alloying elements, including Nb, Ti, and C. Under the ultrahigh cooling rates and steep thermal gradients inherent to LPBF processing, these elements are prone to severe elemental segregation along grain boundaries and melt-pool boundaries. Such segregation promotes the formation of low-melting-point eutectic constituents and brittle Laves phases [56], which subsequently act as preferential crack initiation sites for solidification cracking and liquation cracking [31]. Similarly, in Ti-based alloys, the ultrafast cooling rates suppress the diffusional transformation from the primary *β* phase into the equilibrium *α* + *β* dual-phase microstructure. Instead, a diffusionless phase transformation occurs, yielding a fully brittle acicular *α*′ martensite structure that profoundly deteriorates the macroscopic tensile ductility [50]. Furthermore, in LPBF-processed 304L stainless steels, although ultrafine cellular structures and high-density dislocation networks may enhance strain hardening and facilitate nanotwinning or strain-induced martensitic transformation, the overall strengthening capability remains primarily dependent on conventional solid-solution and dislocation strengthening mechanisms, thereby limiting further improvements in the strength–ductility balance [63].

In comparison, HEAs provide distinct microstructural design opportunities when combined with laser-based AM processes, although their benefits are highly dependent on alloy chemistry, phase stability, and processing conditions. The high configurational entropy, together with enthalpic interactions and elemental partitioning, can promote the formation of simple solid-solution phases in selected HEA systems and reduce the tendency for extensive brittle intermetallic formation. In addition, severe lattice distortion arising from atomic-size and modulus mismatches contributes to intrinsic solid-solution strengthening. For FCC HEAs with relatively low SFE, deformation mechanisms such as twinning-induced plasticity and transformation-induced plasticity can be activated to accommodate plastic strain and enhance work-hardening capacity. Moreover, the submicron cellular substructures, dense dislocation networks, and elemental segregation induced by laser-based AM can serve as effective barriers to dislocation motion and may interact with deformation twins or phase-transformation pathways. Overall, compared with conventional AM alloys, HEAs offer a broader compositional space for tuning phase stability, deformation mechanisms, and AM-induced hierarchical microstructures; however, their performance advantages must be assessed on a composition- and process-specific basis.

## 5. Composition Design for Laser-Based AM HEAs

### 5.1. Printability Criteria

In laser-based additive manufacturing, the printability of HEAs cannot be evaluated using a single criterion because cracking may occur at different stages of the thermal cycle. Therefore, the solidification range, Kou’s cracking susceptibility index (CSI), and strain-age cracking (SAC) index should be distinguished according to the cracking stage and physical mechanism they describe. The solidification range mainly provides a thermodynamic indication of hot-cracking susceptibility; Kou’s CSI further evaluates liquid feeding and grain separation during the terminal stage of solidification; whereas the SAC index describes solid-state cracking associated with precipitation and residual stress during cooling, reheating, or post-processing.

Thermodynamically, the solidification range, also referred to as the freezing range, is defined as the temperature difference between the liquidus and solidus [94,95]. A wider solidification range expands the semi-solid mushy zone, which significantly prolongs the lifetime of vulnerable continuous liquid films and thereby increases the risk of solidification cracking during solidification. Its evaluation requires reliable liquidus and solidus temperatures, which are commonly obtained from CALPHAD calculations, Scheil solidification simulations, or experimental thermal analyses. Compared with more complex cracking susceptibility models, the solidification range is simple and suitable for preliminary screening. For example, in a large-scale assessment of 363 AM alloys, the solidification range model showed the best predictive accuracy for other steel systems, reaching 65.3%, outperforming CSI and Clyne–Davies cracking susceptibility coefficient (CSC) models in this alloy category [94]. In computational design of Ni-based superalloys for AM, the freezing range model can also capture the experimentally observed increase in crack length caused by the addition of alloying elements such as W, Cr, and Re [95]. However, its predictive capability is highly alloy-system-dependent. Because this model only considers the total freezing interval, it cannot distinguish detailed solidification paths. For example, in some Al alloys, grain refiners or early-stage secondary-phase formation can induce a sharp temperature drop at the beginning of solidification, resulting in an L-shaped solidification curve. This artificially enlarges the total solidification range and may overestimate cracking susceptibility. Therefore, in Al alloys, stainless steels, and HEAs, the solidification range generally exhibits lower predictive capability than Kou’s CSI [94]. Furthermore, for multicomponent HEAs, the predictive accuracy of the solidification range may be limited by incomplete thermodynamic databases, strong deviations from equilibrium solidification under LPBF or LDED conditions, local elemental evaporation, and severe microsegregation. Therefore, it should be regarded as a preliminary screening parameter rather than a definitive printability criterion. Minor compositional tailoring can drastically alter this solidification range and, consequently, the printability [96]. For example, in CoCrFeMnNi HEA, the addition of 4 at% TiAl expands the solidification range from 168 °C to 318 °C due to severe Ti segregation along cell and grain boundaries, which promotes solidification cracking during LPBF. However, with further addition of 2.5 at% Cr_3_C_2_, the continuously segregated Ti is transformed into discrete nano-sized TiC particles. This heterogeneous nucleation effectively narrows the solidification range to 274 °C, successfully suppressing hot cracking.

Beyond the macroscopic freezing interval, Kou’s CSI provides a more specific prediction of hot tearing during the terminal stage of solidification. It evaluates the steepness of the modified solidification curve at high solid fractions and is mathematically described as $CSI=\max\left|dT/d\sqrt{f_{s}}\right|$, where T is the temperature and $f_{s}$ is the fraction of the solid [97]. A higher CSI value indicates that thermally induced grain separation is more difficult to compensate for by lateral dendrite growth. This physical competition prevents the closure of interdendritic liquid channels, severely impeding liquid feeding and thereby increasing hot cracking susceptibility. Compared with the solidification range, Kou’s CSI requires more detailed temperature–solid fraction data, which are generally obtained from CALPHAD- or Scheil-based solidification simulations. In terms of predictive capability, the CSI is one of the most effective thermodynamics-based models for AM alloys. It has been reported to outperform the conventional solidification range and CSC models in predicting cracking behavior in AM Al alloys, stainless steels, and HEAs, with predictive accuracies of 70.3%, 87.5%, and 62.2%, respectively [94]. In Nb- and Ta-based refractory alloys evaluated by Varestraint welding tests, the CSI also shows a strong linear correlation with the total crack length, with an $R^{2}$ value of 0.93, and can define quantitative cracking thresholds, such as a CSI < 15,000 K for weldable or printable compositions [98]. Nevertheless, Kou’s CSI still has intrinsic limitations. The model is fundamentally based on thermodynamic solidification behavior and chemical composition, but it does not explicitly account for critical AM-specific physical features, such as grain size, local chemical fluctuations, melt-pool geometry, and rapid solidification kinetics. In particular, solute trapping under extremely high cooling rates can substantially modify segregation behavior and terminal solidification paths, thereby reducing the predictive accuracy of the CSI. Moreover, when different alloy families are analyzed together in a mixed-alloy dataset, the accuracy of the CSI can decrease to approximately 57.6%, which is close to random prediction [94]. This indicates that CSI thresholds must be calibrated for specific alloy systems rather than applied as a universal criterion. For example, a CSI threshold associated with cracking in RHEAs may not be directly transferable to FCC HEAs or conventional superalloys without an alloy-system-specific classification and threshold adjustment.

In addition to liquid-state hot tearing, solid-state strain-age cracking induced by strengthening precipitates serves as another critical printability criterion for laser-built superalloys [84,99] and precipitation-hardened HEAs [83]. Unlike the solidification range and Kou’s CSI, which mainly describe cracking during solidification, the SAC index evaluates cracking susceptibility during solid-state cooling, reheating, or post-processing. Strain-age cracking is fundamentally associated with the interaction between process-induced residual stress and the localized stresses generated by rapid precipitation [100]. It can be quantitatively assessed by the SAC index [95], defined as $SAC={dV}_{\gamma^{\prime }}/dT$, where $V_{\gamma^{\prime }}$ represents the volume fraction of the *γ*′ phase and T is the temperature. A rapid increase in the volume fraction of strengthening precipitates within a narrow temperature interval can generate local volumetric strain and reduce grain-boundary ductility. If the localized strain rate exceeds the intrinsic ductility limit of the material, strain-age cracking can be initiated. In terms of predictive capability, the SAC index is particularly important for high-*γ*′ Ni-based superalloys and precipitation-strengthened HEAs, for which solidification-based models such as the solidification range and CSI cannot fully capture cracking behavior. Compared with traditional empirical formulas based only on the contents of specific elements such as Al, Ti, or Nb, the thermodynamics-based SAC index can more accurately capture crack-length variations caused by microalloying because it directly considers the temperature-dependent evolution of strengthening precipitates [95]. However, its application to multicomponent HEAs remains challenging. Traditional empirical formulas developed for conventional superalloys may fail in newly designed multi-principal-element systems because they cannot capture the complex elemental interactions in HEAs. Moreover, precipitation behavior in HEAs is often governed by coupled effects among sluggish diffusion, severe lattice distortion, local chemical fluctuations, and multiple competing secondary phases. These factors make it difficult to accurately predict the temperature-dependent volume change, precipitation rate, and local strain generated by secondary phases. Therefore, the SAC index should be regarded as a useful but non-universal screening parameter, and its application to HEAs requires alloy-system-specific thermodynamic and kinetic validation.

Overall, the predictive capabilities of these models are complementary rather than interchangeable. The solidification range is simple and useful for preliminary screening, but it may overestimate cracking susceptibility when the solidification path is complex. Kou’s CSI provides a stronger prediction of terminal solidification cracking because it considers the temperature–solid fraction relationship near the final stage of solidification, but its threshold is strongly system-dependent. The SAC index is necessary for predicting precipitation-assisted solid-state cracking, especially in *γ*′-strengthened alloys, but it requires accurate thermodynamic and kinetic descriptions of secondary-phase evolution. Therefore, the composition design of HEAs tailored for laser-based additive manufacturing should not rely solely on a single predictive index or empirical trial-and-error approaches. Instead, the solidification range or CSI should be combined with the SAC index to account for the joint effect of liquid-state hot tearing and solid-state strain-age cracking.

CALPHAD-based calculations using software packages such as Thermo-Calc [101] can provide useful thermodynamic inputs for printability assessment, including liquidus and solidus temperatures, temperature-dependent solid fractions, phase-fraction evolution, and the driving forces for secondary-phase formation. When combined with Scheil solidification simulations, precipitation kinetics analysis, and process-specific thermal-stress evaluation, these criteria can support a more comprehensive assessment of cracking susceptibility in laser-based AM HEAs. However, the reliability of CALPHAD predictions must be carefully interpreted under the non-equilibrium solidification conditions of LPBF and LDED. Conventional equilibrium CALPHAD calculations are useful for identifying phase-stability trends, but they may not accurately capture the actual solidification path during laser-based AM. Steep thermal gradients, rapid cooling, repeated remelting, solute trapping, kinetic undercooling, elemental vaporization, and melt-pool-scale chemical fluctuations can cause the real phase formation behavior to deviate from equilibrium predictions. Therefore, predicted phase fractions, segregation behavior, metastable phases, and precipitation sequences may differ from the as-built microstructures. Scheil simulations can partially account for non-equilibrium solute redistribution and are useful for estimating the solidification range and hot-cracking tendency. Nevertheless, their assumptions of complete liquid mixing, negligible solid-state diffusion, and local equilibrium at the solid–liquid interface are not always valid under the ultrafast solidification conditions of laser-based AM. These limitations are further amplified in HEAs because of their broad compositional space, complex elemental interactions, and limited thermodynamic database coverage for multicomponent systems. Therefore, CALPHAD-based indices, including the solidification range, cracking susceptibility index, and strain-age cracking index, should be calibrated for specific alloy systems rather than treated as universal thresholds. Accordingly, CALPHAD should be regarded as a first-step screening tool rather than a standalone predictor of AM printability. More reliable design strategies require an integrated framework combining thermodynamic prediction, kinetic modeling, melt-pool thermal simulation, thermal-stress analysis, and experimental validation. The summary of design strategies for laser-based AM HEAs is listed in Table 4.

### 5.2. Alloy Systems Reviewed

Among laser-based AM HEAs, alloy systems should be discussed not only as individual compositional cases but also according to their underlying design logic and targeted properties. From this perspective, FCC HEAs are primarily selected for ductility and damage tolerance, RHEAs are developed for high-temperature strength, eutectic or dual-phase HEAs are designed to balance strength and ductility, and precipitation-strengthened HEAs are tailored to improve thermal stability and elevated-temperature performance.

**Table 4:** Design strategies for laser-based AM HEAs.

Design Issue	Processing Route	Observed Defects	Dominant Cause	Alloy Class	Mitigation Mechanism	Key Parameter	Property Impact	Limitation	Example	Ref.
Solidification cracking	LPBF/LDED	Hot cracks along grain/cell boundaries	Wide freezing range, segregation	FCC/dual-phase	Reduces continuous solute-rich liquid films, narrows freezing range	Carbide inoculation, composition tuning	Suppresses hot cracking and improves printability	Excess carbide formation may reduce ductility	Cr_3_C_2_-added Cantor HEA	[96]
Unmelted particles	LPBF/LDED	Unmelted refractory particles and LoF pores	High-melting refractory elements	RHEA	Improves melting of high-melting-point elements	Higher energy density, remelting	Improves ductility	Excessive energy input may cause thermal accumulation, evaporation, or microstructural coarsening	Ti–V–Hf–Nb–Mo	[52]
Low room-temperature ductility	LPBF/LDED	Intergranular microcracks, brittle fracture	BCC brittleness, oxygen segregation	RHEA	Improves grain-boundary cohesion, suppresses oxygen segregation	C/B microalloying, oxygen control	Enhances crack resistance and damage tolerance	Excess C/B or continuous brittle carbide/boride networks may cause interstitial embrittlement	C-added CrMoNbV	[102]
Thermal softening	High-temperature exposure	Strength loss	Dislocation recovery	FCC	Introduces stable strengthening precipitates to compensate for dislocation recovery	Precipitate strengthening	Improves yield strength and thermal stability	Excessive or rapid precipitation may reduce ductility or induce strain-age cracking	L1_2_/B2-strengthened HEA	[55,83]
Anisotropy	LPBF/LDED	Direction-dependent properties	Columnar grains, texture	FCC/EHEA	Refines grains, weakens texture, promotes equiaxed grains	Scan strategy, ultrasound, heat treatment	Reduces mechanical anisotropy	Heat treatment may dissolve beneficial cellular structures or reduce strength	CoCrFeMnNi	[59,90]

Three-dimensional transition-metal FCC HEAs, represented by CoCrFeMnNi- and CoCrFeNi-based alloys, constitute the most widely studied ductility-oriented alloy class for laser-based AM. Thermodynamically driven by high configurational entropy, these alloys typically form a stable single-phase face-centered-cubic structure, endowing them with exceptional ductility and fracture toughness. When fabricated via LPBF, the Cantor alloy develops a hierarchically heterogeneous microstructure featuring unique dislocation cellular networks, which provides the alloy with outstanding thermal stability. Notably, no evident complete recrystallization occurs, even after prolonged annealing at temperatures as high as 900 °C [22]. However, at an annealing temperature of 1000 °C, these cellular substructures progressively disintegrate due to the extensive annihilation of entangled dislocations and the continuous dissolution of elemental segregation at the cell boundaries. In contrast, the low-angle grain boundaries (LAGBs) exhibit superior thermal stability and remain effective up to 1000 °C, quantitatively providing a 17.13% contribution to the overall yield strength [103]. Conversely, Cantor alloys processed via LDED typically exhibit coarser columnar grains compared to those produced by LPBF, primarily owing to the larger laser spot size and thicker deposition layers associated with the LDED process [62]. To mitigate this issue, introducing high-intensity ultrasonic vibration during the LDED process can effectively refine the average equiaxed grain size from 140 to 44 μm. This profound grain refinement is mainly driven by acoustic cavitation-induced dendrite fragmentation and enhanced local supercooling. Consequently, governed by the classic Hall–Petch relationship, the ultrasonic-assisted LDED CoCrFeMnNi HEA achieves a ~17% increase in tensile yield strength without a discernible compromise in ductility [90].

For applications requiring superior high-temperature strength, RHEAs provide a distinct design pathway, but their alloy design inherently involves a tradeoff between printability and elevated-temperature performance. These alloys are composed of high-melting-point elements, such as Nb, Mo, Ta, W, V, Zr, and Hf, and generally form BCC solid-solution structures. The phase stability and strengthening mechanisms in these alloys are fundamentally governed by severe lattice distortion, which arises from the significant atomic radii mismatch among the constituent elements [38]. This unique structural characteristic endows RHEAs with superior yield strength, exceptional hardness, and good thermal stability at elevated temperatures [52]. However, the same compositional features that contribute to high-temperature strength also make laser-based AM challenging. The high melting points of refractory elements increase the risk of incomplete melting, LoF defects, and unmelted particles, whereas the intrinsic room-temperature brittleness of BCC RHEAs limits their ability to accommodate AM-induced residual thermal stresses. Consequently, RHEAs are highly susceptible to solidification cracking [25]. In addition to thermal stress and intrinsic BCC brittleness, interstitial elements, particularly oxygen, nitrogen, and carbon, play a critical role in determining the printability and mechanical performance of laser-based AM RHEAs. Oxygen contamination is generally detrimental because metallic powders with high specific surface areas can readily absorb oxygen from the environment. During rapid solidification and repeated thermal cycling, oxygen tends to segregate along grain boundaries, reducing grain-boundary cohesive strength and promoting intergranular microcracking under AM-induced residual stresses [25]. Similarly, although small amounts of oxygen and nitrogen can provide interstitial solid-solution strengthening, an excessive interstitial content may lead to severe interstitial embrittlement and a dramatic loss of plastic deformability [52]. Nevertheless, the effects of these interstitials are not universally harmful. In specially designed RHEAs, controlled oxygen doping has been reported to form ordered oxygen complexes, which can modify dislocation shearing behavior, promote the transition from planar slip to wavy slip, facilitate double cross-slip and dislocation multiplication, and thereby simultaneously improve strength and ductility [104]. Therefore, the role of oxygen and nitrogen in RHEAs is highly dependent on their concentration, segregation behavior, and whether they remain in solid solution or form ordered complexes or brittle compounds. To mitigate the cracking susceptibility of RHEAs while retaining their high-temperature strength, microstructural engineering and process optimization have been proposed. The Ti_1.5_Nb1Ta_0.5_Zr_1_Mo_0.5_ RHEA fabricated by the LPBF process exhibits a hierarchically heterogeneous microstructure characterized by a unique submicron cellular network. Within this structure, Ti is preferentially segregated to the cell walls, whereas Ta and Nb are predominantly enriched within the cell interiors. During the initial stages of plastic deformation, these cell walls act as potent barriers that effectively restrict dislocation mobility, thereby substantially enhancing the yield strength. As the applied stress and structural deformation increase, extensive dislocation pile-ups occur adjacent to the cell boundaries. Consequently, the primary role of the cell walls dynamically transitions from impeding dislocation glide to actively absorbing and storing the accumulating dislocations. This dynamic absorption mechanism significantly suppresses detrimental localized strain bursts and promotes highly homogeneous deformation within the grains, which synergistically contributes to a clear improvement in the overall ductility [104]. The addition of 1 wt% B into the equiatomic MoNbTaTiZr powder promotes the in situ precipitation of XB_2_-type borides along the grain boundaries and interdendritic regions during the LPBF process. These borides effectively restrict grain growth and promote a more homogeneous strain distribution, synergistically enhancing the microhardness and suppressing crack formation [105]. However, this strategy also illustrates the tradeoff between printability and strengthening. Discrete borides can improve grain-boundary stability and crack resistance, but excessive or continuous brittle boride networks may reduce ductility and damage tolerance. Furthermore, during the LDED process, high-melting-point Nb and Mo elements are prone to forming LoF defects or unmelted particles under insufficient energy input conditions. These defects act as severe stress concentrators, promoting crack initiation and eventually causing premature brittle fracture. Implementing a remelting strategy or increasing the laser energy input can effectively diminish the fraction of processing defects in Ti_41_V_27_Hf_13_Nb_13_Mo_6_ RHEA. Consequently, this shifts the dominant deformation and damage mechanism from a cracking-governed mode to a microvoid-dominated coalescence mode, thereby resulting in outstanding tensile performance and enhanced ductility [52]. Among interstitial elements, carbon microalloying is one of the most effective grain-boundary engineering strategies for suppressing microcracking in laser-based AM RHEAs. The microalloying of 1 at% C into Cr_25_Mo_25_Nb_25_V_25_ RHEA can significantly suppress oxygen segregation along the grain boundaries, thereby enhancing intergranular cohesion and mitigating oxygen-induced embrittlement [102]. Furthermore, the localized enrichment of carbon near the grain boundaries effectively narrows the solid–liquid coexistence zone, which substantially reduces contraction stresses and constrains the initiation of solidification cracking. In addition to its grain-boundary effect, carbon dissolved in the BCC matrix can also provide interstitial solid-solution strengthening through interactions with dislocations. It is crucial to note, however, that excessive carbon addition can induce the extensive precipitation of brittle carbides, which paradoxically exacerbates thermal cracking and proves highly detrimental to the overall ductility of the alloy [102]. Furthermore, Al addition can reduce the density of RHEAs and contribute to a lightweight alloy design. In the LDED-processed Al_0.8_Nb_0.5_Ti_2_V_2_Zr_0.5_ lightweight RHEA, Al not only reduces the alloy density but also contributes to the solid-solution strengthening through strong interactions with the other constituent elements. Al and Zr tend to segregate into interdendritic regions, promoting the formation of Laves phases. The main strengthening contributions in this RHEA arise from severe lattice distortion, solid-solution strengthening, and second-phase strengthening by Laves phases [47].

Dual-phase HEAs provide another important design route by combining the plastic deformability of FCC phases with the high strength of BCC/B2 phases. These alloys predominantly comprise 3D transition metals that form an FCC matrix, judiciously alloyed with BCC-stabilizing elements such as Al, Ti, W, and Mo. The incorporation of BCC stabilizers shifts the thermodynamic equilibrium of the system from a single-phase to a dual-phase region, driving phase separation or eutectic reactions during solidification and thereby generating a heterogeneous dual-phase microstructure. Among these alloys, the AlCoCrFeNi_2.1_ EHEA is the most extensively investigated system. In this EHEA, the soft FCC phase provides plastic deformability and strain hardening, whereas the hard BCC/B2 phase accommodates high local stresses and contributes to yield strength and hardness. Therefore, the mechanical performance and crack resistance of dual-phase HEAs are strongly governed by eutectic spacing, phase fractions, interfacial coherency, and lamellar orientation during LPBF/LDED thermal processes. The eutectic spacing is particularly sensitive to the rapid solidification conditions of laser-based AM. Compared with conventional casting, the high cooling rates in LPBF and LDED suppress long-range elemental diffusion and refine cellular or lamellar dual-phase structures to the submicron or nanoscale range. Specifically, the extreme cooling rates inherent to LPBF can significantly suppress elemental partitioning between the constituent phases and generate a highly refined FCC(L1_2_)/BCC(B2) nano-lamellar structure [106]. The reduction in eutectic spacing increases the density of phase boundaries, which act as strong barriers to dislocation motion. Consequently, geometrically necessary dislocations accumulate at FCC/BCC interfaces, generating pronounced hetero-deformation-induced hardening and back stress. This interfacial strengthening mechanism can substantially enhance yield and tensile strength while maintaining ductility through a sustained strain-hardening rate [75]. However, excessively refined nanolamellar structures may also restrict the plastic zone under high triaxial-stress conditions, such as fracture-toughness testing. In such cases, cracks may propagate rapidly along phase boundaries because large-scale plastic deformation and extrinsic toughening mechanisms are limited [107]. The phase fraction between FCC(L1_2_) and BCC(B2) phases is another critical factor. During LPBF or LDED, the VED, scanning speed, cooling rate, and elemental evaporation, particularly Al evaporation, can modify local phase stability and alter the FCC/BCC ratio. A higher FCC fraction generally improves ductility because the FCC phase contains multiple slip systems and can accommodate plastic deformation through dislocation slip, planar slip, and SFs formation. In contrast, the BCC/B2 phase provides higher initial strength but is more prone to strain localization and microcrack initiation. Therefore, an optimized FCC/BCC phase fraction is essential for achieving strength–ductility synergy. During deformation, microcracks tend to initiate preferentially in the harder and more brittle BCC/B2 phase. A sufficient fraction of ductile FCC phase can blunt crack tips, accommodate local strain, and suppress rapid crack propagation, thereby improving crack resistance and delaying premature brittle fracture [106]. Interfacial coherency and orientation relationships further determine whether the soft and hard phases can deform cooperatively. Rapid solidification during AM can promote semi-coherent FCC/BCC interfaces with specific orientation relationships, such as the Kurdjumov–Sachs relationship. These semi-coherent interfaces are beneficial for strength because they impede dislocation glide and promote dislocation accumulation. At the same time, favorable interfacial coherency can provide channels for dislocation transmission from the FCC phase into the BCC/B2 phase once the local stress reaches a critical level. This interfacial dislocation transfer promotes co-deformation between the soft and hard phases, stabilizes plastic flow, and delays early fracture [106]. However, under complex triaxial-stress states, semi-coherent interfaces may also become weak links if plastic compatibility between the two phases is insufficient. Severe mechanical incompatibility can induce local stress concentration, interfacial decohesion, and crack propagation along phase boundaries [107]. Thus, interfacial characteristics can enhance strength and work hardening under tensile loading, but they must be carefully controlled to avoid interface-dominated fracture. Lamellar orientation also plays an important role because LPBF and LDED impose strong directional thermal gradients. During laser-based AM, eutectic colonies or lamellar structures tend to grow along the heat-flow direction or building direction, resulting in crystallographic texture and geometric anisotropy. When the loading direction is parallel to the lamellar growth direction, adjacent eutectic colonies can exhibit better deformation compatibility and more uniform stress distribution. The aligned FCC/BCC interfaces effectively block dislocation motion, promote interfacial dislocation pile-up, and enhance hetero-deformation-induced hardening, leading to superior strength and ductility. Conversely, when loading is perpendicular to the lamellar structure, the less favorable alignment of phases can promote local stress concentration and premature cracking. Crack resistance is also orientation-dependent: if crack propagation is parallel to continuous phase boundaries, cracks may advance rapidly along weak interfaces, with limited crack deflection or bridging. In contrast, when lamellar orientation promotes dislocation storage and crack-tip blunting, ductile fracture can be maintained under uniaxial tensile loading [53]. Moreover, microstructural evolution is highly sensitive to laser processing parameters. In LPBF, increasing the VED transitions the microstructure of the AlCoCrFeNi_2.1_ HEA from a cellular grain morphology into a well-defined layered eutectic structure with finer, more stable lamellae [44]. In LDED, applying an interlayer pause strategy offers a robust approach to tailor the eutectic morphology. It has been demonstrated that a 60 s interlayer pause can achieve an approximately 40% refinement in the lamellar thickness [75]. Overall, the design logic of laser-based AM dual-phase HEAs is not only to combine soft FCC and hard BCC/B2 phases, but also to optimize eutectic spacing, phase fraction, interfacial coherency, and lamellar orientation. These parameters collectively determine dislocation storage, load transfer, strain partitioning, crack-tip blunting, and interfacial decohesion, thereby controlling the final balance among strength, ductility, and crack resistance.

Another important design strategy for laser-based AM HEAs is precipitation strengthening, which is mainly aimed at improving thermal stability, resistance to thermal softening, and elevated-temperature strength. In contrast to single-phase FCC HEAs, whose AM-induced dislocation cellular structures may gradually recover during thermal exposure, precipitation-strengthened HEAs can maintain higher strength through thermally stable secondary phases. For example, the strategic incorporation of Al and Ti into CoCrFeNi-based HEAs promotes the formation of L1_2_-strengthened microstructures. In the LDED-processed Ni_45_Co_20_Fe_10_Cr_10_Al_7.5_Ti_7.5_ HEA, the *γ* + *γ*′ phase structure improves microstructural stability and provides effective precipitation strengthening, resulting in a room-temperature yield strength of 514 MPa and an ultimate tensile strength exceeding 605 MPa, although the tensile elongation is limited to approximately 4% [73]. Similarly, LPBF-processed precipitation-strengthened HEAs with high Al and Ti contents have been developed to provide intrinsic hot-cracking resistance and ultrahigh strength, demonstrating the potential of composition design for simultaneously controlling printability and the strengthening response [83].

## 6. Mechanical Properties

The mechanical properties of HEAs fabricated via laser-based additive manufacturing are fundamentally governed by their underlying deformation mechanisms, which are inherently dictated by the crystal structure, SFE, dislocation dynamics, and phase constitution. In FCC HEAs, plastic deformation is predominantly accommodated by dislocation glide, twinning-induced plasticity, and transformation-induced plasticity. This combined deformation behavior typically endows FCC HEAs with an outstanding strength–ductility combination. Conversely, the deformation mechanisms in BCC HEAs and RHEAs are primarily controlled by restricted dislocation mobility and severe lattice distortion. Consequently, these alloys often exhibit ultrahigh yield strengths, albeit at the expense of room-temperature ductility. Furthermore, the specific laser processing parameters and the unique hierarchically heterogeneous microstructures intrinsically induced by the rapid solidification of laser-based AM processes profoundly modulate the overall mechanical performance.

### 6.1. Room Temperature Properties

**Figure 4:**
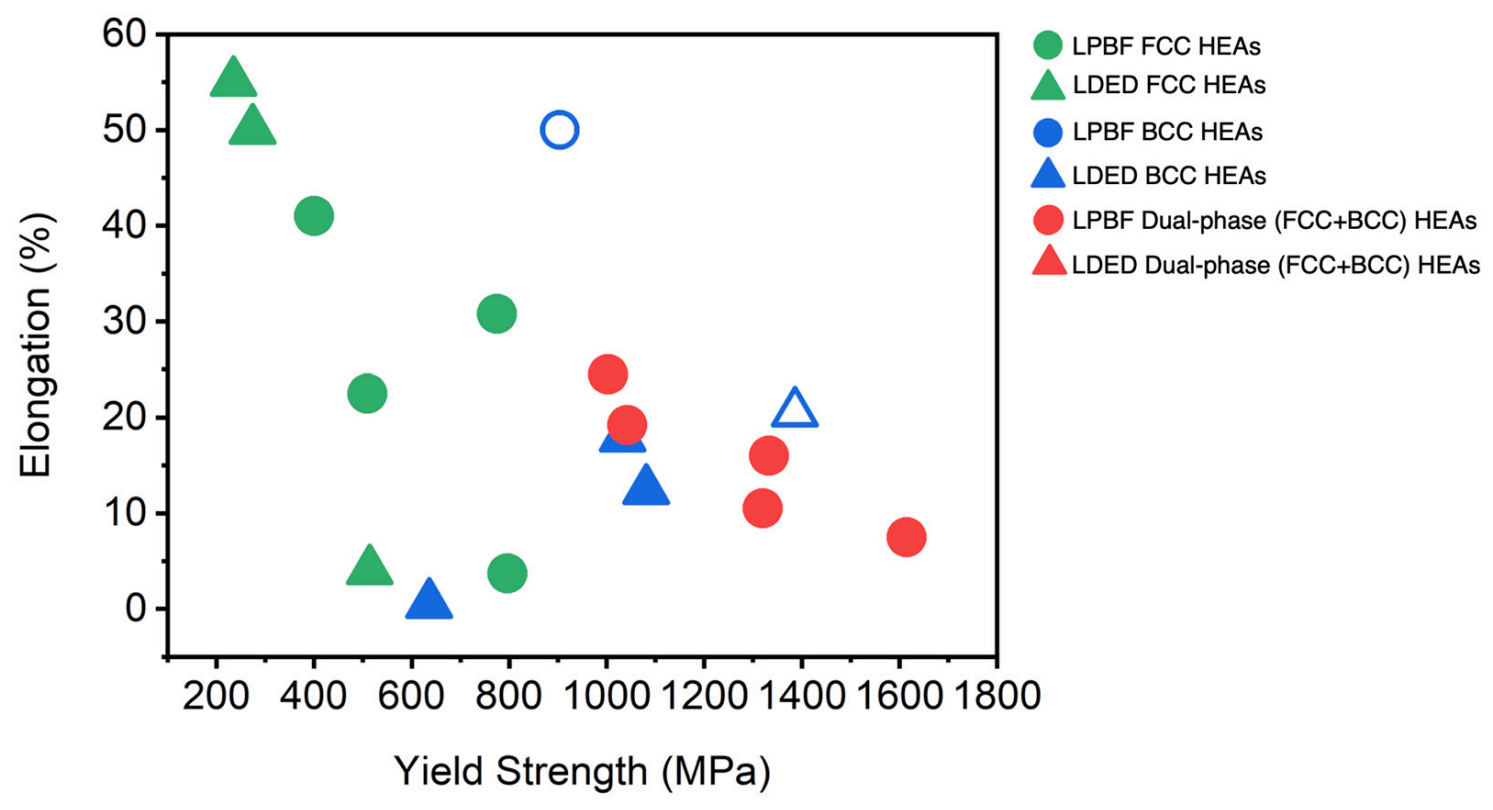
Property map of as-built laser-based AM HEAs under room-temperature mechanical test [10,47,52,60,73,76,90,104,106,107,112,114]. Solid symbols indicate tensile properties, and open symbols indicate compressive properties. Different colors/shapes represent different alloy categories.

Fundamentally, 3D-transition metal FCC HEAs exhibit outstanding ductility and robust strain-hardening capacity; however, their room-temperature yield strengths are generally inferior to those of BCC HEAs. Laser-based additive manufacturing provides a promising pathway to overcome this strength–ductility trade-off by inducing hierarchically heterogeneous microstructures. For instance, under optimized laser processing parameters, an in situ alloyed non-equiatomic FeCoCrNi HEA can develop a highly refined grain structure populated with high-density dislocations and novel hybrid crystal-amorphous precipitates. This unique microstructural architecture collectively contributes to a yield strength of 400 MPa coupled with an exceptional tensile elongation exceeding 41% [60]. Beyond compositional tuning, process interventions such as ultrasonic-assisted LDED can further promote microstructural refinement. In the CoCrFeMnNi HEA, the acoustic cavitation effect during LDED significantly refines the equiaxed grains, resulting in a 17% improvement in yield strength without a discernible compromise in ductility [90], which is 44 MPa higher than the as-cast sample under same strain rate [108]. The LPBF CoCrFeNi HEA also exhibits superior tensile performance compared to the as-cast CoCrFeNi HEA, which usually presents the yield strength within the range of 200 to 300 MPa [70,108,109,110]. Furthermore, minor alloying additions or particle reinforcements into HEA matrices can profoundly tailor microstructural evolution and phase formation during AM. Specifically, introducing Ti into a CoCrFeNi matrix exacerbates lattice distortion owing to its larger atomic radius, thereby amplifying solid-solution strengthening. Concurrently, Ti acts as a heterogeneous nucleation site, triggering a columnar-to-equiaxed transition during the LPBF process, while precipitating a nanoscale Ni_3_Ti network along the subgrain boundaries. Consequently, the LPBF-processed CoCrFeNiTi_0.3_ HEA exhibits a remarkable yield strength increase from 509 MPa to 796 MPa, albeit at the expense of tensile ductility [70]. The strategic incorporation of Al and Ti into the CoCrFeNi HEA system effectively promotes the formation of a *γ* + *γ*′ phase structure within the multicomponent FCC HEA matrix during the LDED process. This microstructural evolution significantly enhances the thermal stability, coarsening resistance, and overall mechanical strength of the alloy. Specifically, the in situ precipitation of the *γ*′ phase acts as a potent strengthener. Consequently, the LDED-processed Ni-Co-Fe-Cr-Al-Ti HEA achieves a substantially improved room-temperature yield strength of 514 MPa and an ultimate tensile strength exceeding 605 MPa, while retaining a tensile elongation of approximately 4% [73]. Similarly, the incorporation of NbC into the Al_3.6_Co_27.2_Cr_19_Fe_18_Ni_26_Ti_5.5_Zr_0.01_Si_0.3_ HEA refines the cell structures. The MC carbides serve as heterogeneous nucleation sites, driving a substantial grain size reduction from 27 μm to 7 μm at a 5 wt% NbC addition. Driven by the combined effects of grain and cell refinement, and carbide dispersion strengthening, the yield strength notably increases from 630 MPa for 0 wt% NbC to 677 MPa for 1 wt% NbC, while successfully maintaining an excellent ductility of 30.6% [21]. The mechanical properties are summarized in Table 5, together with the property map in Figure 4.

Refractory HEAs typically exhibit yield strength and high-temperature thermal stability. However, their practical applications are inherently hindered by room-temperature brittleness. Consequently, the room-temperature mechanical performance of RHEAs is predominantly evaluated via compression testing rather than tensile loading. To overcome this strength–ductility trade-off, microstructural engineering via additive manufacturing has proven highly effective. For instance, in the LPBF-processed TiNbTaZrMo RHEA, the uniquely induced submicron cellular structure acts as a potent barrier to constrain dislocation mobility during the early stages of deformation. As the applied stress increases, these cell walls dynamically absorb and store the accumulating dislocations, thereby promoting a highly homogeneous strain distribution. This self-accommodating behavior culminates in an exceptional compressive yield strength of 904 MPa and a remarkable compressive strain exceeding 50% without fracture [104], which effectively improves the ductility performance compared to the as-cast sample [111]. In a parallel advancement, recent studies have demonstrated that significant tensile ductility can also be achieved in RHEAs through rigorous process optimization. By tailoring the laser parameters during the LDED process, the fraction of deleterious processing defects in the Ti_41_V_27_Hf_13_Nb_13_Mo_6_ RHEA can be substantially mitigated. This critical defect reduction shifts the dominant damage mechanism from a cracking-governed mode to a microvoid-dominated coalescence mode, thereby yielding an outstanding tensile yield strength of 1033 MPa coupled with an unprecedented tensile elongation of 17.9% at room temperature [52]. Furthermore, compositional tailoring, such as strategic light-element microalloying, provides a combined strategy for further property enhancement. The incorporation of Al into RHEAs not only effectively reduces the overall density of the LDED-processed alloy but also facilitates the formation of a hierarchical microstructure. Specifically, the Al-modified RHEA exhibits a refined bimodal grain-size distribution—comprising alternating equiaxed and columnar grains—within a BCC matrix, accompanied by the in situ precipitation of Al-Zr-V-rich C14_Laves phases along the grain and subgrain boundaries. Driven by the synergistic effects of this bimodal architecture and potent second-phase strengthening, the room-temperature compressive yield strength of the alloy reaches an impressive 1386 MPa, alongside a substantial plastic strain of 20.5% [47]. The mechanical properties are summarized in Table 5, coupled with the property map in Figure 4.

Dual-phase HEAs effectively integrate the intrinsic advantages of a soft FCC phase and a hard BCC phase, thereby yielding a synergistic strength–ductility balance. For instance, AlCoCrFeNi_2.1_ EHEA fabricated via LPBF exhibits a highly refined nanolamellar architecture consisting of alternating FCC and BCC phases. This unique microstructure affords an exceptionally high tensile yield strength of 1320 MPa coupled with a uniform elongation of 10.5% [107]. Furthermore, through the precise optimization of VED during the LPBF process, the AlCoCrFeNi_2.1_ EHEA can attain a tailored yield strength of 1042 MPa and an enhanced ductility of 19.2%. These values substantially outperform those of the as-cast counterpart tested under identical conditions, which exhibits a yield strength of merely 550 MPa and an elongation of 15.8% [106]. In the AlCoCrFeNi_2.1_ EHEA processed by LDED, pronounced location-dependent variations in tensile performance are observed along the building direction. Specifically, the bottom region exhibits superior ductility but lower strength, whereas the top region displays elevated strength at the expense of ductility. Notably, the middle region achieves an optimal strength–ductility synergy. This distinctive mechanical gradient is fundamentally governed by the synergistic evolution of phase proportions and microstructural morphologies across different building elevations. In the bottom region, the pronounced cyclic reheating from subsequently deposited layers promotes the transition of BCC to FCC, resulting in a high-volume fraction of the soft FCC phase characterized by coarse columnar grains. Conversely, as the deposition height increases, the reduced thermal cycling effect leads to a significant increase in the hard BCC phase fraction. More importantly, the microstructure in the middle and top regions transitions into a highly refined lamellar FCC/BCC structure. Particularly in the middle region, this fine lamellar heterostructure effectively triggers hetero-deformation-induced hardening while permitting coordinated plastic co-deformation between the soft and hard phases, thereby simultaneously sustaining high strength and preserving remarkable ductility [112]. Mechanistically, the ultrafine nanolamellar structure in AlCoCrFeNi_2.1_ EHEA imposes a severe spatial confinement on dislocation mobility. This effect, superimposed with the high density of printing-induced pre-existing dislocations, significantly contributes to the overall strengthening. During plastic deformation, the applied load is efficiently transferred from the softer FCC phase to the harder BCC phase, ensuring progressive yielding and sustained strain hardening. Driven by these combined mechanisms, the LPBF-processed alloy can achieve a yield strength of 1333 MPa alongside an elongation of ~16%, presenting a more than twofold strength increase over the as-cast state (510 MPa yield strength and ~16% ductility) [76]. This microstructural design strategy is also highly versatile; a similar nanolamellar structure can be successfully realized in the Ni_40_Co_20_Fe_10_Cr_10_Al_18_W_2_ EHEA by the LPBF process. Compared to its as-cast equivalent, the additively manufactured variant demonstrates a remarkable 747 MPa improvement in yield strength while preserving a comparable tensile elongation of ~15% [76,113]. The mechanical properties are summarized in Table 5, coupled with the property map in Figure 4.

**Table 5:** Room-temperature engineering stress–strain properties of as-built laser-based AM HEAs under tensile and compressive loading.

	Crystal Structure	Density (%)	Grain Size	Process	Build Orientation	Test Condition	Strain Rate (s^−1^)	Specimen Geometry	Yield Strength (MPa)	Tensile Strength (MPa)	Fracture Strain (%)	Ref.
CoCrFeNi	FCC	99.5	38.5 μm	LPBF	0°	Tensile	3.33 × 10^−4^	Dog-bone-shaped, gauge dimension of 25 × 3 × 1.5 mm^3^	509	649	22.5	[70]
CoCrFeNiTi_0.3_	FCC	99.5	30.4 μm	LPBF	0°	Tensile	3.33 × 10^−4^	Dog-bone-shaped, gauge dimension of 25 × 3 × 1.5 mm^3^	796	905	3.7	[70]
CoCrFeNi + SS316L	FCC	-	9 μm	LPBF	0°	Tensile	1 × 10^−3^	Dog-bone-shaped, gauge dimension of 10 × 2.5 × 3.2 mm^3^	400	561	41	[60]
CoCrFeMnNi	FCC	-	6 μm	LPBF	0°	Tensile	1 × 10^−3^	-	775	923	31	[114]
CoCrFeMnNi	FCC	99.57	44 μm	LDED	0°	Tensile	1 × 10^−4^	-	274	-	50	[90]
CoCrFeMnNi	FCC	99.57	140 μm	LDED	0°	Tensile	1 × 10^−4^	-	235	-	55	[90]
AlCoCrFeNiTi	FCC	-	116 μm	LDED	0°	Tensile	-	ASTM E8/E8M-18 standard	514	605	4	[73]
TiNbTaZrMo	BCC	99.82	4–9 μm	LPBF	90°	Compressive	1 × 10^−3^	Φ 6.5 × 6.5 mm^2^	904	2500	50	[104]
Ti_41_V_27_Hf_13_Nb_13_Mo_6_	BCC	-	~130 μm	LDED	0°	Tensile	1 × 10^−3^	gauge dimensions of 4 × 1.5 × 1 mm^3^	636	-	0.5	[52]
Ti_41_V_27_Hf_13_Nb_13_Mo_6_	BCC	-	~150 μm	LDED	0°	Tensile	1 × 10^−3^	gauge dimensions of 4 × 1.5 × 1 mm^3^	1033	1093	17.9	[52]
Ti_41_V_27_Hf_13_Nb_13_Mo_6_	BCC	-	~140 μm	LDED	0°	Tensile	1 × 10^−3^	gauge dimensions of 4 × 1.5 × 1 mm^3^	1081	1111	12.4	[52]
Al_0.8_Nb_0.5_Ti_2_V_2_Zr_0.5_	BCC	-	44 μm for fine equiaxed grains, 101 μm for columnar grains	LDED	90°	Compressive	1 × 10^−3^	Φ 4 × 6 mm^2^	1386	1590	20.5	[47]
AlCoCrFeNi_2.1_	Dual	>99	BCC: 51 nm FCC: 181 nm	LPBF	0°	Tensile	1 × 10^−3^	Dog-bone-shaped, gauge dimension of 12 × 2.5 × 1.5 mm^3^	1320	1590	10.5	[107]
AlCoCrFeNi_2.1_	Dual	>99.9	BCC: 89 nm FCC: 128 nm	LPBF	90°	Tensile	5 × 10^−4^	Dog-bone-shaped, gauge dimension of 6 × 2 × 2 mm^3^	1042	1380	19.2	[106]
AlCoCrFeNi_2.1_	Dual	-	-	LDED	0°	Tensile	1 × 10^−3^	Dog-bone-shaped, gauge dimension of 8 × 2.5 × 1 mm^3^	554	954	24	[112]
AlCoCrFeNi_2.1_	Dual	-	-	LDED	0°	Tensile	1 × 10^−3^	Dog-bone-shaped, gauge dimension of 8 × 2.5 × 1 mm^3^	637	1091	18.1	[112]
AlCoCrFeNi_2.1_	Dual	-	-	LDED	0°	Tensile	1 × 10^−3^	Dog-bone-shaped, gauge dimension of 8 × 2.5 × 1 mm^3^	721	1105	13	[112]
AlCoCrFeNi_2.1_	Dual	>99.5	BCC: 64 nm FCC: 151 nm	LPBF	0°	Tensile	2 × 10^−4^	Dog-bone-shaped, gauge dimension of 8 × 2 × 1 mm^3^	1333	1640	16	[76]

Note: “-” indicates that the corresponding information was not reported in the cited references.

### 6.2. High-Temperature Performance

High-temperature performance of laser-based AM HEAs should not be evaluated only from short-term tensile or compressive tests. For structural applications, oxidation resistance, creep resistance, phase stability, and microstructural changes during thermal exposure are also critical. Therefore, this section first summarizes the short-term elevated-temperature mechanical properties and then discusses the long-term stability issues that govern high-temperature service reliability.

FCC HEAs generally exhibit excellent room-temperature ductility and strain-hardening capability, but their yield strength often decreases substantially at elevated temperatures. Precipitation hardening, microalloying [115], and AM-induced submicron microstructures are commonly used to improve high-temperature performance. FeCoCrNi HEAs exhibit an outstanding combination of yield strength and ductility at room temperature, owing to their high-density dislocation networks [116]. However, when the testing temperature exceeds 600 °C, the dislocation network is eliminated, resulting in thermal softening. Furthermore, nanoclustering has been reported to be detrimental to ductility [117], which will cause elemental segregation along grain boundaries and grain boundary decohesion. Therefore, the FeCoCrNi HEA presents a yield strength of 150 MPa and a 6.1% elongation under an 800 °C tensile test [118].

Refractory HEAs fundamentally exhibit outstanding high-temperature strength, primarily owing to severe lattice distortion-induced solid-solution strengthening. Additive manufacturing techniques can further elevate these high-temperature capabilities through unique microstructural evolutions. For instance, the rapid thermal cycles inherent to the LDED process introduce a high density of intrinsic dislocations of $6.26\times 10^{13}$ m^−2^ into the non-equiatomic Nb_40_Ta_25_Ti_15_Hf_15_Zr_5_ RHEA. While screw dislocations are predominantly annihilated at 1000 °C, edge dislocations exhibit exceptional thermal stability. These preserved intrinsic edge dislocations provide substantial local slip resistance by hindering the motion of newly activated dislocations, thereby maintaining an outstanding strain-hardening capacity. Consequently, this AM-processed RHEA achieves an impressive ultimate tensile strength of around 497.3 MPa and a uniform elongation of ~6.8% at 1000 °C, representing substantial increases of ~37.8% and ~61.9%, respectively, over its fully recrystallized as-cast counterpart [57]. In addition to intrinsic defects, interstitial microalloying presents another robust pathway for high-temperature strengthening. By introducing 1 at% C into the (Cr_25_Mo_25_Nb_25_V_25_)_99_C_1_ RHEA, solidification cracking and oxygen segregation along the grain boundaries during the LDED process are effectively suppressed. At elevated temperatures, this alloy benefits from a potent dislocation pinning effect within both dendritic and interdendritic regions, which is synergistically facilitated by severe lattice distortion, local chemical fluctuations, solute atoms, and complex dislocation interactions. As a result, its compressive yield strength maintains a remarkably high level of 787 MPa, even at 1000 °C [102]. Furthermore, dynamic microstructural evolution at elevated temperatures can additionally counteract thermal softening. The LDED-processed Al_0.8_Nb_0.5_Ti_2_V_2_Zr_0.5_ RHEA exhibits a high compressive yield strength of 940 MPa and a fracture strain of 25.7% at 600 °C. As the testing temperature increases to 800 °C, despite a reduction in yield strength to 450 MPa, the alloy demonstrates extraordinary deformability, sustaining over 50% compressive strain without fracture. The retention of such a respectable yield strength at 800 °C is largely attributed to the dynamic precipitation and growth of the intermetallic C14_Laves phase, which efficiently accommodates localized stresses at high temperatures and partially compensates for the strength degradation induced by solid-solution softening [47].

**Figure 5:**
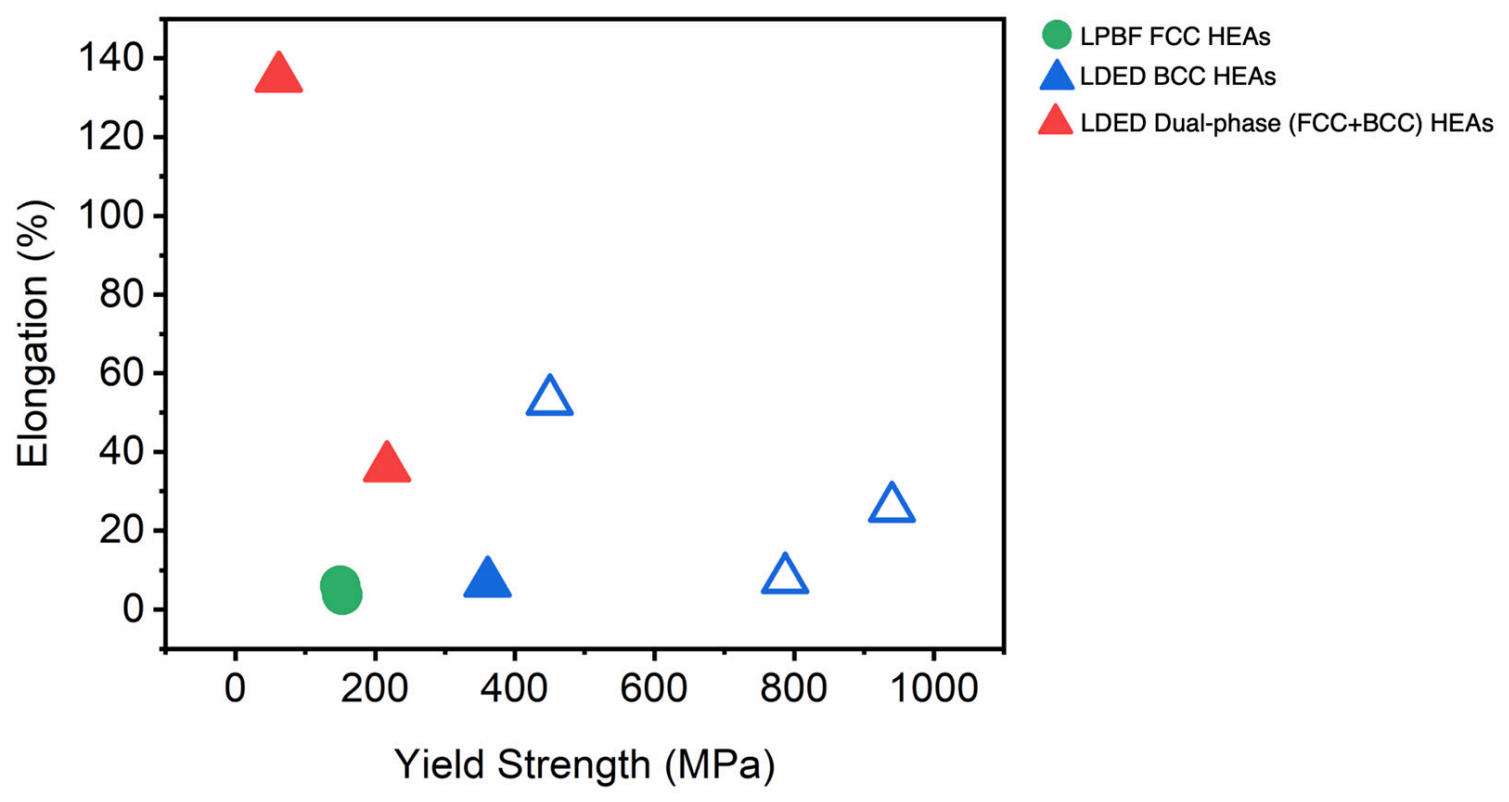
Property map of as-built laser-based AM HEAs under high-temperature mechanical test [47,57,68,69,102,118]. Solid symbols indicate tensile properties, and open symbols indicate compressive properties. Different colors/shapes represent different alloy categories.

The synergistic advantages of FCC and BCC phases endow dual-phase HEAs with extraordinary mechanical properties under elevated-temperature conditions. For instance, the LDED-processed Fe_36_Ni_35_Al_17_Cr_10_Mo_2_ EHEA features a hierarchical microstructure comprising a reticulated dual-phase dendritic network, microscale heterogeneous grains, and nano-scale BCC precipitates. This unique microstructural architecture provides an excellent room-temperature yield strength of 640 MPa, while maintaining a respectable ultimate tensile strength of 217 MPa, even at an elevated temperature of 800 °C. Furthermore, at elevated temperatures, the delicate dual-phase lamellar exhibits enhanced coordinated deformation, effectively suppressing the nucleation and propagation of microcracks at the phase boundaries and ensuring a ductile fracture mode [69]. Similarly, the LDED-processed Ni_32_Co_30_Cr_10_Fe_10_Al_18_ EHEA demonstrates outstanding room-temperature tensile properties, driven by the coordinated co-deformation of its FCC and B2 dual-phase structure. However, during tensile testing at an extreme high temperature of 982 °C, this EHEA experiences a severe thermal softening effect, leading to a precipitous drop in ultimate tensile strength to approximately 62 MPa. Nevertheless, benefiting from the extensive dynamic recrystallization at this temperature, the alloy accommodates significant plastic flow, thereby presenting an exceptionally large uniform elongation prior to fracture [68]. The high-temperature mechanical properties are summarized in Table 6, coupled with the property map in Figure 5.

**Table 6:** High-temperature engineering stress–strain properties of as-built laser-based AM HEAs under tensile and compressive loading.

	Crystal Structure	Density (%)	Grain Size	Process	Build Orientation	Test Condition	Strain Rate (s^−1^)	Specimen Geometry	Yield Strength (MPa)	Tensile Strength (MPa)	Fracture Strain (%)	Ref.
FeCoCrNi	FCC	-	-	LPBF	90°	Tensile/800 °C	5 × 10^−3^	Φ 5 mm with gauge length of 25 mm	150	-	6.1	[118]
FeCoCrNi	FCC	-	-	LPBF	90°	Tensile/600 °C	5 × 10^−3^	Φ 5 mm with gauge length of 25 mm	153	-	3.7	[118]
Nb_40_Ta_25_Ti_15_Hf_15_Zr_5_	BCC	-	-	LDED	0°	Tensile/1000 °C	1 × 10^−3^	-	361	497	6.8	[57]
(Cr_25_Mo_25_Nb_25_V_25_)_99_C_1_	BCC	-	46 μm	LDED	0°	Compressive/1000 °C	1 × 10^−3^	Φ 2 × 4 mm^2^	787	1377	7.7	[102]
Al_0.8_Nb_0.5_Ti_2_V_2_Zr_0.5_	BCC	99.57	44 μm for fine equiaxed grains, 101 μm for columnar grains	LDED	90°	Compressive/600 °C	1 × 10^−3^	Φ 4 × 6 mm^2^	940	1558	25.7	[47]
Al_0.8_Nb_0.5_Ti_2_V_2_Zr_0.5_	BCC	99.57	44 μm for fine equiaxed grains, 101 μm for columnar grains	LDED	90°	Compressive/800 °C	1 × 10^−3^	Φ 4 × 6 mm^2^	450	510	~53	[47]
Fe_36_Ni_35_Al_17_Cr_10_Mo_2_	Dual	-	-	LDED	90°	Tensile/800 °C	1 × 10^−3^	Dog-bone-shaped, gauge dimension of 12.5 × 3 × 2 mm^3^	~217	217	~36	[69]
Ni_32_Co_30_Cr_10_Fe_10_Al_18_	Dual	-	-	LDED	90°	Tensile/982 °C	1 × 10^−3^	-	~62	62	~135	[68]

Note: “-” indicates that the corresponding information was not reported in the cited references.

In addition to short-term mechanical properties, oxidation resistance is a key factor controlling the high-temperature applicability of AM HEAs. The refined microstructures produced by LPBF or LDED can improve oxidation behavior by increasing the density of grain boundaries, which serve as fast diffusion pathways for protective oxide-forming elements. For example, LPBF-processed CrMnFeCoNi HEA with refined grains and abundant grain boundaries can promote the rapid formation of a continuous and dense Cr_2_O_3_ scale while suppressing the formation of less protective spinel oxides. As a result, its mass gain at 900–1100 °C is lower than that of its cast counterpart [119]. For Al-containing HEAs and RHEAs, Al plays a particularly important role because it promotes the formation of a stable *α*-Al_2_O_3_ protective layer. In LDED-processed Al_0.15_(CrMoTaTi)_0.85_ RHEA, careful control of the Al content enables the formation of an external Al_2_O_3_ layer, even at 1300 °C, which helps overcome the poor oxidation resistance commonly observed in many RHEAs [120]. Surface treatments can further improve oxidation resistance. Laser shock peening, for example, introduces compressive residual stress and surface grain refinement, which can reduce the oxidation rate and suppress oxide-scale spallation during high-temperature exposure [121].

Creep resistance is another important requirement for high-temperature service. The sluggish diffusion effect alone is not sufficient to ensure high creep resistance; stable nanoscale obstacles, subgrain boundaries, and grain-boundary engineering are also needed. In LPBF-processed CrMnFeCoNi HEAs, interstitial carbon addition can promote in situ carbide formation. These nanocarbides, together with AM-induced subgrain networks, reduce the minimum creep rate and improve creep resistance compared with conventionally processed counterparts [122]. In LPBF-processed Ti_1.5_Ta_0.5_NbZrMo_0.5_ RHEA tested at 923–1023 K, continuous precipitates and dislocation tangles form near grain boundaries during creep. These heterogeneous structures hinder further dislocation glide and cutting, leading to a lower minimum creep rate [123]. For precipitation-strengthened HEAs, post-processing can also be used to improve creep life. For example, serrated grain boundaries produced by heat treatment provide geometric interlocking against grain-boundary sliding. This strategy increases the creep rupture life at 650 °C/650 MPa from 0.58 h to 274 h [20].

Phase stability during thermal exposure is closely related to the stability of AM-induced substructures. The high-density dislocation cell networks formed during LPBF are thermodynamically unstable at elevated temperatures. In some LPBF HEAs, these cell structures begin to degrade at approximately 400 °C and almost disappear after exposure near 1000 °C. However, low-angle grain boundaries can retain their strengthening effect up to 1000 °C, providing better thermal stability than dislocation cell walls [103]. In situ-formed nanoscale oxides, such as Mn-rich oxides distributed along cellular or subgrain boundaries, can further stabilize the microstructure by pinning grain boundaries. This pinning effect suppresses abnormal grain growth and recrystallization during thermal exposure, thereby preserving microstructural integrity at high temperatures [22].

Long-term thermal exposure can also lead to irreversible microstructural changes that alter the strength–ductility balance. At very high temperatures, such as 1000–1200 °C, intermetallic precipitates in BCC RHEAs may dissolve or coarsen. In Al_0.5_Nb_1.25_Ta_1.25_TiZr RHEA, Al-Zr-rich precipitates originally located near grain boundaries can coarsen or dissolve during thermal exposure, leading to the formation of precipitate-free zones. These precipitate-free zones weaken grain-boundary strength and promote intergranular fractures during high-temperature deformation, reducing fracture resistance [86]. More generally, thermal exposure causes dislocation annihilation, recovery, residual-stress relaxation, and partial recrystallization in AM HEAs. These processes often reduce yield strength and hardness but may improve tensile ductility by relieving residual stresses and reducing lattice distortion. Therefore, high-temperature performance should be interpreted as a balance between short-term strength, oxidation resistance, creep resistance, phase stability, and microstructural degradation during thermal exposure.

### 6.3. As-Built State Fatigue and Fracture

Since simple monotonic tensile and compressive tests cannot adequately represent the dynamic mechanical responses of structural components under service conditions, fatigue and fracture properties are essential for establishing a comprehensive process–structure–property relationship for industrial applications. Generally, fatigue crack initiation in laser-based AM FCC HEAs is highly sensitive to processing defects, such as LoF voids, keyhole pores, and unmelted powder particles. Two major defect morphologies are commonly considered: highly spherical gas pores and irregular defects, including LoF defects, subsurface keyhole pores, and unmelted powder particles. Quantitative defect statistics indicate that pores, solid inclusions such as unmelted particles, and grain sizes in AM alloys often follow a log-normal size distribution [124]. Most pores remain within a subcritical size range; for example, more than 90% of pores may exhibit equivalent diameters below 30 μm [71]. However, fatigue failure is usually governed by the largest defects in the distribution tail rather than by the average pore size. As spherical gas pores can exhibit very high sphericity, their relatively smooth morphology produces lower local stress concentration compared with irregular defects. In contrast, LoF defects and subsurface keyhole pores possess sharp edges, elongated geometries, and poor local metallurgical continuity, which substantially amplify the local cyclic stress field. Therefore, even when their number density is lower than that of spherical gas pores, these irregular defects are far more detrimental to fatigue life [125]. Their projected area perpendicular to the loading direction and their sharp morphology effectively increase the local stress intensity, reduce the threshold for fatigue crack initiation, and promote premature crack growth under cyclic loading.

Accordingly, fatigue crack initiation in laser-based AM HEAs is governed by the competition between defect-driven initiation and intrinsic slip-driven initiation. When pronounced internal or near-surface defects are present, fatigue cracks preferentially initiate from LoF defects, unmelted powder particles, or keyhole pores, particularly those located within approximately 200 μm from the surface. These defects may further coalesce during cyclic loading, forming a large effective initial crack and significantly shortening fatigue life [125]. In contrast, when critical surface and subsurface defects are removed by machining or polishing, or when the internal defect population is sufficiently low, the crack initiation mechanism can shift toward intrinsic microstructural damage. Under such conditions, fatigue cracks tend to nucleate at persistent slip bands (PSBs) or local microstructural heterogeneities caused by cyclic strain localization [114,125,126]. Residual stresses introduced during laser-based AM further influence fatigue crack initiation and propagation [127]. The steep thermal gradients and repeated heating–cooling cycles generally generate tensile residual stresses near the surface and along the scanning direction, while compressive residual stresses may remain in the interior of the component. Tensile residual stresses increase the local mean stress and facilitate crack openings, thereby accelerating fatigue crack growth. Conversely, compressive residual stresses promote crack closure and reduce the effective driving force for crack propagation. Therefore, controlling residual stresses is essential for improving fatigue resistance.

In addition to crack initiation, fatigue crack growth resistance and fracture toughness are closely associated with AM-induced microstructural anisotropy and deformation mechanisms. Owing to the columnar grain structures commonly formed during laser-based AM, the threshold stress intensity factor range, $\Delta K_{th}$, can be direction-dependent. When the crack path intersects a high density of columnar grain boundaries, subgrain boundaries, and cellular structures, crack propagation becomes more tortuous, thereby increasing fatigue crack growth resistance. In particular, when the loading direction is perpendicular to the build direction, propagating cracks are forced to cut across dense columnar grains and subgrain boundaries, which can enhance $\Delta K_{th}$ and improve resistance to fatigue crack propagation [71].

The fracture toughness of laser-based AM HEAs is also affected by intrinsic and extrinsic toughening mechanisms. In metastable FCC HEAs, intense stress concentration near the crack tip can activate TRIP or TWIP, such as the *γ* to *ε* martensitic transformation or nanoscale deformation twinning. These mechanisms absorb deformation energy and provide sustained local strain hardening, thereby resisting further crack propagation [124]. In addition, heterogeneous microstructures containing high-density dislocation cellular structures, precipitates, and grain boundaries can promote crack deflection and crack branching. The resulting tortuous crack path increases the effective crack propagation distance and promotes roughness-induced crack closure and grain bridging, which reduce the effective stress intensity at the crack tip and enhance fracture resistance [127].

In single-phase FCC HEAs, such as LPBF-processed CoCrFeNi HEA, deformation twins can be activated under high cycle fatigue at a maximum stress of 450 MPa (R = 0.1). These fatigue-induced twins accommodate plastic strain and contribute to macroscopic cyclic softening, yielding a fatigue strength of 170 MPa [126]. To further enhance fatigue resistance, introducing in situ-formed oxides presents a highly effective strategy. For instance, in LPBF-processed CoCrFeMnNi HEAs, in situ-formed Mn_2_O_3_ nanoparticles distributed along the subgrain and grain boundaries provide substantial Orowan strengthening. This effect, coupled with a high-density dislocation network that generates back-stress, effectively constrains the formation of persistent slip bands. Consequently, the high cycle fatigue limit reaches 570 MPa, approximately twice that of its cast counterpart [114].

Alternatively, triggering metastable phase transformations offers an excellent defect-tolerant strategy. The metastable Fe_40_Mn_20_Co_20_Cr_15_Si_5_ HEA exhibits a high cycle fatigue strength of 325 MPa (R = −1), even in the presence of printing pores. This robust fatigue performance is attributed to the sustained work hardenability induced by the transformation-induced plasticity from the *γ* (FCC) to *ε* (HCP) phase, followed by deformation twinning within the newly formed *ε* phase [124], which outperforms typical AM TWIP steels (~300 MPa) [128]. Similarly, a hierarchical microstructure comprising an FCC matrix reinforced with both coherent L1_2_ and B2 incoherent precipitates in the LPBF-processed Al_11.94_Cr_17.76_Fe_18.71_Ni_47.67_V_3.87_O_0.05_ HEA yields a high fatigue strength of ~300 MPa (R = 0.1). During cyclic loading, the interaction between mobile dislocations and B2 precipitates promotes the formation of new dislocation cells, which helps release local stress concentrations. As fatigue deformation accumulates, these cell walls dynamically evolve into low-angle grain boundaries, effectively preventing highly localized strain [71]. Conversely, BCC RHEAs typically suffer from intrinsic room-temperature brittleness, making them highly susceptible to intergranular cracking during macroscopic elastic deformation, which fundamentally limits their room-temperature fatigue evaluations. This brittleness, combined with their high sensitivity to unmelted refractory particles, oxygen segregation, irregular defects, and tensile residual stresses, fundamentally limits systematic room-temperature fatigue and fracture evaluations. Therefore, future studies on laser-based AM RHEAs should place greater emphasis on quantitative defect statistics, fatigue crack growth behavior, $\Delta K_{th}$, fracture toughness, and residual-stress management to establish more reliable damage-tolerant design criteria.

## 7. Post-Processing and Heat Treatment

The rapid cooling rates and repeated thermal cycles inherent to laser-based additive manufacturing processes typically induce high residual stresses, processing defects, elemental microsegregation, and hierarchical microstructures characterized by cellular subgrains and high-density dislocation entanglements. Therefore, post-processing treatments are essential for reducing residual stresses and healing defects, as well as controlling phase evolution and tailoring the final mechanical performance of laser-based AM HEAs. To better distinguish their functions, these strategies can be broadly categorized into thermal treatments, including stress relief, homogenization, solution treatment, and aging; thermomechanical densification treatments, such as HIP; and low-temperature or surface mechanical treatments, such as cryogenic treatment and laser shock peening. Each treatment targets different microstructural features and provides distinct advantages, but may also introduce potential risks, including thermal softening, grain coarsening, precipitate embrittlement, phase instability, or surface damage. The post-processing strategies for laser-based AM HEAs are summarized in Table 7. It should be noted that the temperature, duration, and cooling conditions listed in Table 7 represent typical processing windows rather than universal parameters. The optimal post-processing route should be selected according to the alloy chemistry, phase stability, defect population, residual-stress state, and AM-induced microstructural features that need to be retained.

Low-to-medium-temperature heat treatments are generally conducted for stress relief, aiming to preserve the unique AM-induced microstructures while relaxing residual thermal stresses primarily through creep-dominated mechanisms. With an optimized stress-relief temperature, residual stresses can be effectively eliminated, significantly enhancing the material’s damage tolerance and reducing its susceptibility to cracking [129]. Furthermore, intermediate-temperature annealing provides the necessary thermal activation energy to facilitate localized phase transformations. While single-phase FCC HEAs typically do not undergo significant phase transformations during annealing [116], intermediate temperatures in specific HEA systems can trigger the precipitation of secondary phases. For instance, heat treating the Al_0.75_Mn_0.25_CoCrFeNi EHEA at 850 °C promotes the precipitation of a (Cr, Fe)-enriched *σ* phase [78], whereas the (FeCoNi)_86_Al_7_Ti_7_ HEA exhibits dual L1_2_ and L2_1_ precipitates under similar thermal conditions [55].

Conversely, elevated-temperature annealing, often serving as homogenization and solution heat treatments (typically ranging from 1000 °C to 1200 °C), is primarily utilized to eradicate elemental segregation and fully homogenize the matrix [116,130,131]. High-temperature thermal exposure effectively dissolves brittle intermetallic compounds, reverting the alloy to a simple solid-solution state [78,132], which is a critical prerequisite for subsequent aging treatments aimed at uniformly dispersing strengthening precipitates [132]. For example, the brittle *σ* phase induced in the Al_0.75_Mn_0.25_CoCrFeNi EHEA can be completely dissolved at 1150 °C, concurrently promoting the precipitation of B2 nanoclusters that contribute to an exceptional strength–ductility synergy [78]. From a microstructural perspective, high temperatures facilitate full recrystallization, transforming the initial columnar grains and molten pool boundaries into equiaxed grains [116,133]. During this process, the ultrafine nanolamellar architectures in dual-phase HEAs undergo severe coarsening and morphological fragmentation [130]. Concurrently, the annihilation of AM-induced cellular structures and high-density dislocation networks leads to a macroscopic thermal softening effect, which substantially improves tensile ductility but inevitably compromises the final yield strength and hardness [116].

**Table 7:** Summary of post-processing strategies for laser-based AM HEAs.

Post-Treatment	Condition	Main Purpose	Affected Alloy Types	Microstructural Effect	Benefit	Risk	Anticipated Property Trade-Off	Ref.
Stress relief	Low-to-medium temperature, typically 650–700 °C for 2–4 h, followed by air cooling or furnace cooling.	Residual stress reduction	Residual-stress-sensitive AM HEAs	Preserves cells, partial stress relaxation	Crack resistance, ductility improvement	Strength loss	Improves ductility and damage tolerance but may reduce yield strength and hardness.	[129]
Homogenization/Solution treatment	High-temperature annealing, typically 1000–1150 °C for 1–4 h. Cooling depends on alloy stability; rapid cooling or water quenching is preferred when brittle precipitation during cooling is a concern.	Segregation removal	HEAs with severe elemental segregation and alloys requiring chemical homogenization before further aging	Dissolves cell segregation	Improves chemical uniformity and ductility	Grain coarsening, strength loss	Improves uniform plasticity and microstructural stability, but often lowers yield strength and hardness.	[78,116,130,131,132,133]
aging	Intermediate temperature, commonly 600–800 °C for 4–30 h, usually followed by air cooling.	Precipitate strengthening	Precipitation-strengthened HEAs	L1_2_/B2/Laves formation	High strength	SAC/brittleness	Strongly improves strength, but may reduce ductility if precipitation is excessive.	[55]
HIP	High temperature with high isostatic pressure, typically 1000–1150 °C, 100–150 MPa Ar, for 2–4 h. Cooling rate depends on the HIP system.	Pore closure	Broadly applicable to AM HEAs, especially defect-sensitive HEAs	Densification/recrystallization	Fatigue/fracture	Thermal softening	Maximizes densification and fatigue resistance but often sacrifices yield strength and hardness.	[134,135,136,137]
Cryo-treatment	Immersion in liquid nitrogen at −196 °C, typically 24–96 h, followed by rewarming to room temperature.	Stress tailoring	Metastable FCC HEAs, dual-phase HEAs	Phase transformation/grain refinement/residual stress reversal	Strength–ductility synergy	Phase instability	Can improve strength and ductility together in suitable metastable alloys, but benefits are limited in highly stable systems.	[58,138,139,140,141]
Laser shock peening	RT surface mechanical treatment using pulsed laser-induced shock waves	Conversion of surface tensile residual stress into compressive residual stress, and mitigation of surface-defect sensitivity	HEAs requiring surface treatment	Near-surface gradient deformation layer with ultrafine/nanograins and compressive residual stress	Improves surface hardness and crack-initiation resistance	Limited to surface and subsurface regions; excessive peening may cause surface damage or distortion in thin parts	Enhances surface strength and fatigue resistance but might cause surface damage.	[133,138]

Additionally, to concurrently address AM-induced processing defects, HIP is widely used as an effective post-treatment strategy for defect-sensitive laser-based AM alloys. During HIP, the simultaneous application of elevated temperature and high isostatic pressure, typically 100–300 MPa, promotes localized plastic flow, creep deformation, and diffusion bonding. These mechanisms facilitate the closure and healing of internal gas pores, LoF defects, and hot cracks, thereby substantially improving densification and damage tolerance [134,135,136]. For instance, in the LPBF-processed CoCuFeMnNITi_0.13_ HEA, HIP increases the relative density from 94.0% to 99.8% by healing internal gas pores and liquation-induced hot cracks [135]. Similarly, in the LPBF-processed CoCrFeMnNi HEA, HIP successfully eradicates microvoids and unmelted particles. Benefiting from the extreme thermal exposure during HIP, the initial columnar grains undergo full recrystallization accompanied by the formation of annealing twins, which virtually eliminates the microstructural and mechanical anisotropies inherent to the AM process [137]. Because pores and LoF defects usually serve as preferential fatigue crack initiation sites, defect closure by HIP can also markedly improve fatigue resistance. For example, in LPBF-processed Hastelloy X, HIP increases the four-point bending fatigue limit from below 450 MPa to approximately 650 MPa, demonstrating the strong benefit of defect elimination for fatigue-critical AM components [136]. However, the beneficial effect of defect healing must be balanced against the loss of AM-induced strengthening. The unique high strength of LPBF-processed alloys often originates from non-equilibrium cellular subgrain structures, high-density dislocation networks, elemental microsegregation, and refined melt-pool-related microstructures generated by ultrahigh cooling rates. Conventional HIP treatments are usually conducted at high temperatures, such as 1000–1150 °C for several hours, which can erase these beneficial hierarchical features [137]. During HIP, cellular subgrain structures and dislocation networks may be annihilated, while the original columnar grains can recrystallize into coarser equiaxed grains accompanied by annealing twins. For example, in LPBF-processed Hastelloy X, ultrafine primary dendrites with diameters below 1 μm can coarsen to approximately 10–20 μm after HIP [136]. Similarly, in LPBF-processed CoCrFeMnNi HEA, HIP eliminates microstructural anisotropy through full recrystallization, but the yield strengths of the horizontal and vertical specimens decrease by approximately 50% and 41%, respectively [137]. Therefore, although HIP improves ductility, fracture toughness, and fatigue life by removing crack initiation sites, it can simultaneously reduce yield strength and hardness through thermal softening, dislocation annihilation, and grain coarsening. A further limitation of conventional HIP is the slow cooling rate, commonly associated with furnace cooling, which may provide sufficient time for undesirable phase transformations during cooling. In AM Al_0.85_CoCrFeNi HEA, rapid solidification initially suppresses the formation of detrimental equilibrium phases; however, the slow cooling rate during HIP, around 5 K/min or lower, allows the alloy to remain within the critical precipitation range of approximately 600–960 °C for an extended period. This thermal exposure promotes the precipitation of the brittle *σ* phase, resulting in severe embrittlement and premature tensile fracture [134]. In dual-phase Al_0.6_CoCrFeNi HEA, HIP can also induce abnormal coarsening of Ni–Al-rich hard B2 precipitates along phase boundaries and grain boundaries, which severely deteriorates tensile ductility and promotes early brittle fracture [112]. Therefore, HIP should not be regarded as universally beneficial for laser-based AM HEAs. Instead, its temperature, holding time, pressure, and cooling rate must be carefully optimized according to the alloy chemistry, defect population, phase stability, and desired balance between defect tolerance and AM-induced strengthening.

Deep cryogenic treatment (DCT) has emerged as a non-destructive post-processing strategy for tailoring residual stresses and defect structures in laser-based AM HEAs. However, its effectiveness strongly depends on the phase stability of the alloy, and DCT should not be regarded as universally applicable to all laser-processed HEAs [58,138,139,140,141]. The most favorable alloy systems for DCT are metastable FCC or dual-phase HEAs with low SFE and limited FCC-phase stability, such as the non-equiatomic Fe_80−*x*_Mn_x_Co_10_Cr_10_ alloys, particularly the laser-based AM-processed dual-phase Fe_50_Mn_30_Co_10_Cr_10_ HEA. The ultralow temperature during DCT significantly reduces the SFE. Concurrently, the localized thermal stress and micro-plastic deformation, induced by the inconsistent volumetric contraction among grains, provide a robust thermo-mechanical driving force. This strongly facilitates the TRIP effect, driving the martensitic transformation from the FCC *γ* matrix to the HCP *ε* phase. This TRIP-assisted response increases the fraction of the harder HCP phase, introduces dense FCC/HCP-phase boundaries, and fragments the original coarse FCC grains. For example, prolonged DCT can increase the HCP-phase fraction by approximately 2.2 times and reduce the average grain size by about 58%, indicating that metastable HEAs can benefit from both phase transformation and microstructural refinement during cryogenic exposure [58,138]. In contrast, thermodynamically stable single-phase FCC HEAs and medium-entropy alloys, such as equiatomic CoCrFeMnNi and CrCoNi, respond to DCT through different mechanisms. These alloys maintain high FCC-phase stability, and no evident phase transformation may occur, even after long DCT durations such as 120 h. Therefore, the cryogenic thermal stresses in these stable FCC systems are mainly accommodated by localized visco-plastic deformation rather than by TRIP. When the local thermal stress exceeds the critical stress for defect generation or twin nucleation, high-density dislocations, SFs, and nanoscale deformation twins can be introduced into the matrix [140,141]. These defects, together with the compressive residual stresses generated during cooling and rewarming, can improve yield strength and crack resistance. Nevertheless, the scale of microstructural modification is generally more limited than that in metastable HEAs, and the average grain size may remain nearly unchanged after DCT. Therefore, DCT is most effective for metastable HEAs that are capable of cryogenic TRIP, whereas in stable FCC HEAs or medium-entropy alloys, its benefits are mainly derived from dislocation/twinning-mediated strengthening and residual-stress redistribution. During the rewarming stage back to room temperature, the irreversible plastic deformation that occurred at cryogenic temperatures restricts free thermal expansion. This mismatch directly triggers a complete stress reversal, transforming the AM-induced tensile residual stresses into highly beneficial compressive residual stresses (CRSs). During subsequent tensile loading, this pronounced CRS partially compensates for the applied external stress, thereby effectively suppressing microcrack initiation and propagation. Accordingly, DCT can improve the strength–ductility balance of suitable laser-based AM HEAs, but its processing window should be selected according to alloy metastability, SFE, phase constitution, and the susceptibility to TRIP or TWIP activation.

Laser shock peening (LSP) is an advanced surface-strengthening and cold-working post-treatment for laser-based AM HEAs. During LSP, high-energy pulsed laser irradiation generates intense shock waves on the material surface, imposing ultrahigh-strain-rate plastic deformation in the near-surface region. Its primary function is to convert harmful tensile residual stresses into beneficial compressive residual stresses and to generate a gradient deformation layer near the surface. This layer is commonly characterized by grain refinement, dislocation multiplication, SFs, and deformation twins [133]. In metastable HEAs, LSP can further activate strain-induced martensitic transformation, providing an additional strengthening pathway through FCC-to-HCP transformation [138]. The effectiveness of LSP has been demonstrated in laser-based AM CrMnFeCoNi-based HEAs. In an LDED-processed CrMnFeCoNi HEA, direct LSP increased the surface microhardness and introduced a compressive residual stress field, while annealing followed by LSP produced a thicker plastically affected layer and a deeper compressive stress profile. This improvement occurs because prior annealing reduces the initial dislocation network, relaxes tensile residual stress, and improves surface plastic deformability, allowing the subsequent LSP shock wave to induce more extensive plastic deformation. In this case, the combined annealing + LSP treatment generated a gradient microstructure containing ultrafine grains, high-density mechanical twins, and slip bands, thereby improving the yield strength, ultimate tensile strength, and ductility simultaneously. This indicates that LSP is particularly effective when the surface layer has sufficient plastic accommodation capability before shock loading [133]. For metastable HEAs, LSP can be further combined with deep cryogenic treatment (DCT) to overcome the limited treatment depth of LSP. In LPBF-fabricated Fe_50_Mn_30_Co_10_Cr_10_ HEA, DCT first promotes internal stress redistribution, micro-plastic deformation, and partial martensitic transformation, whereas subsequent LSP introduces a surface gradient heterogeneous structure with refined grains, increased dislocation density, and a higher HCP-phase fraction. The combined DCT + LSP treatment transforms the initial surface tensile residual stress into compressive residual stress and enhances the surface hardness. The resulting mechanical improvement is associated with the combined effects of hetero-deformation-induced hardening, TRIP/TWIP activation, and compressive residual stress [138]. Nevertheless, LSP also has several important limitations. Because the shock wave attenuates rapidly during inward propagation, its effect is mainly confined to the surface and subsurface regions, typically from several tens to several hundreds of micrometers depending on the alloy and processing parameters. Therefore, LSP cannot effectively eliminate deep internal defects or excessive tensile residual stresses in the core region of thick components. In addition, the strengthening response of LSP strongly depends on the initial plasticity and deformation resistance of the material. In as-built AM HEAs containing dense immobile dislocation networks, the high deformation resistance may restrict shock-induced plastic deformation, resulting in a shallow affected layer and limited strengthening. Therefore, LSP should be considered a complementary surface-strengthening treatment rather than a substitute for bulk defect-healing treatments such as HIP. For practical design, LSP is often more effective when combined with suitable pretreatments, such as annealing to improve plastic deformability or DCT to tailor the bulk residual stress state before surface strengthening.

**Figure 6:**
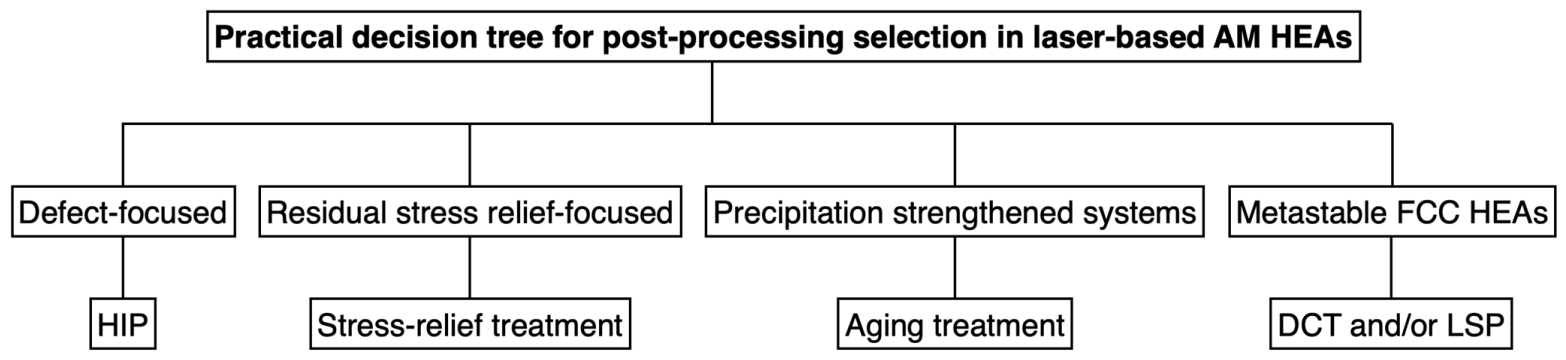
Practical decision tree for post-processing choices.

Overall, the effects of post-processing treatments on residual stress relaxation and defect mitigation are strongly dependent on the alloy class because each alloy system has different defect types, phase stability, and strengthening mechanisms. For RHEAs, conventional stress-relief heat treatment may reduce crack-driving residual stresses; however, it is generally insufficient to heal microcracks or LoF defects generated during AM. HIP is therefore more effective for intrinsically brittle RHEAs. For example, in LPBF-fabricated WMoNbTa, HIP at 1350 °C under 150 MPa for 4 h can nearly eliminate internal micropores and connected microcracks, release residual stresses, promote recrystallization, and suppress oxygen segregation at grain boundaries [25]. These effects improve compressive strength and fracture strain, although the annihilation of high-density dislocations may reduce microhardness. In contrast, the roles of DCT and LSP in RHEAs remain less established and are mainly expected to modify residual stresses or near-surface damage rather than heal internal defects. For EHEAs such as AlCoCrFeNi_2.1_, post-processing is highly sensitive to heat-treatment temperature because their strength–ductility balance relies on refined FCC/BCC or FCC/B2 lamellar structures. Low-temperature stress relief can relax residual stresses and reduce dislocation density, but it may also lower the yield strength by removing AM-induced dislocation strengthening. At higher annealing temperatures, strong recrystallization may fragment the continuous dual-phase lamellae and weaken the cooperative deformation between the soft and hard phases [130]. Therefore, although HIP may help reduce defects in EHEAs, excessive thermal exposure should be carefully avoided because lamellar coarsening and phase fragmentation can reduce interfacial strengthening. DCT and LSP may provide additional stress redistribution or surface strengthening, but they should be selected only when the phase stability and lamellar morphology can be retained. For precipitation-strengthened HEAs, heat treatment is the main post-processing route. Solution treatment followed by aging can dissolve undesirable brittle phases and promote the controlled precipitation of coherent L1_2_, B2/L2_1_, carbides, or Laves-related phases. In LPBF-fabricated (FeCoNi)_86_Al_7_Ti_7_ HEA, annealing and aging can generate nanoscale, coherent L1_2_ precipitates together with submicron L2_1_/B2 precipitates, which delay slip-band propagation and improve fatigue resistance [55]. In LDED Ni-rich HEAs, multi-step solution and aging treatments can partially dissolve blocky Laves phases, transform them into finer particles, and introduce dense nanoscale L1_2_ precipitates, thereby improving strength while retaining useful ductility [30]. However, retained tensile residual stresses and rapid precipitation may increase the risk of strain-age cracking; therefore, stress relief before aging is often necessary. HIP may be useful when internal defects are severe, but it should be followed by solution and aging treatments to restore precipitation strengthening. For stable single-phase FCC HEAs, high-temperature annealing and HIP can close pores and relax residual stresses, but they often erase AM-induced cellular substructures and dislocation networks. This causes clear strength reduction despite improved ductility and defect tolerance [133,135]. DCT provides a lower-temperature alternative. In AM CoCrFeMnNi HEA, cryogenic contraction can redistribute residual stresses and introduce dislocations or nanotwins without significant grain coarsening [141]. LSP is also useful as a surface mechanical treatment. After annealing, LSP can compensate for strength loss by introducing ultrafine surface grains, high-density mechanical twins, and deep compressive residual stresses, increasing yield strength while maintaining strain-hardening capacity [133]. Overall, post-processing selection should be based on the alloy class, defect population, residual-stress state, phase stability, and the AM-induced strengthening features that should be preserved. Based on these alloy-class-dependent responses, Figure 6 summarizes a practical decision tree for selecting post-processing routes for laser-based AM HEAs.

## 8. Challenges and Future Perspectives

### 8.1. Current Limitations

Although LPBF and LDED provide effective routes for fabricating geometrically complex HEA components and tailoring hierarchical microstructures, the key limitations of laser-based AM HEAs are strongly alloy-class dependent. Therefore, current challenges should be prioritized according to both their severity and the specific alloy systems involved.

Among the reported alloy classes, RHEAs face the most severe printability challenges. Because RHEAs contain high-melting-point elements such as W, Mo, Ta, and Nb, the large differences in melting temperature, density, and thermal properties among the constituent elements make complete melting difficult during rapid laser processing. Under insufficient laser energy input, high-melting-point elements, especially Mo- and Nb-rich particles, may remain partially unmelted. These unmelted particles disrupt microstructural homogeneity and serve as strong stress concentrators, promoting microvoid coalescence, crack nucleation, and premature brittle fracture [52]. In addition, RHEAs are highly sensitive to oxygen contamination. Oxygen adsorbed on powder surfaces or retained in the processing chamber can segregate to grain boundaries during solidification and subsequent thermal cycling. This grain-boundary oxygen enrichment reduces boundary cohesion and promotes intergranular embrittlement, which is further aggravated by the high residual tensile stresses generated during LPBF or LDED [25].

Precipitation-strengthened HEAs represent another highly crack-sensitive alloy class. In these systems, high contents of Al, Ti, Nb, or other precipitation-forming elements are introduced to promote L1_2_ or B2 strengthening. However, the same alloying elements also increase the risk of solidification and liquation cracking during laser-based AM. During the final stage of solidification, segregation of solute elements can promote the formation of low-melting-point constituents. These constituents may form continuous liquid films along grain boundaries, which cannot accommodate the tensile stresses generated during cooling shrinkage. As a result, hot tearing and grain-boundary liquation cracks can readily develop [82]. Moreover, strain-age cracking is a major solid-state cracking risk in precipitation-strengthened systems. During post-solidification cooling or subsequent heat treatment, rapid precipitation of *γ*′ or related strengthening phases can generate high local transformation and volumetric stresses. When these stresses are superimposed on AM-induced residual stresses, cracking can occur, even in the solid state [31,142]. Elemental vaporization in precipitation-strengthened alloys is also highly dependent on alloy chemistry rather than being governed only by laser energy input. In multicomponent alloys, each element exhibits a distinct boiling point, vapor pressure, and enthalpy of vaporization. Therefore, elements with low boiling points or high saturated vapor pressures are preferentially lost from the melt pool under high laser energy input. Volatile strengthening elements such as Al and Ti are essential for the formation of *γ*′ precipitates. Their selective evaporation can locally reduce the *γ*′-forming ability, weaken precipitation strengthening, and degrade high-temperature strength. Thus, compositional deviation caused by vaporization should be interpreted as a chemistry-dependent metallurgical instability rather than simply as a processing defect.

Dual-phase HEAs are attractive because their coupled FCC/BCC phase structures can provide high strength while retaining useful ductility. However, their performance is highly sensitive to the laser VED. If the VED is too low, LoF defects and interlayer delamination can form. If the VED is too high, keyhole porosity and preferential vaporization of low-boiling-point elements may occur. This problem is strongly chemistry dependent. For example, in the AlCoCrFeNi_2.1_ EHEA system, Al has a lower boiling point and lower vaporization enthalpy than Co, Cr, Fe, and Ni. Therefore, increasing VED can cause selective Al evaporation and measurable Al loss. Since Al is a key BCC/B2 stabilizer and contributes to lattice distortion strengthening, excessive Al loss can reduce the volume fraction and stability of the hard BCC/B2 phase. Consequently, the intended eutectic phase balance is disturbed, and the yield strength and tensile strength may decrease with increasing laser energy input [44]. In addition, the mechanical response of EHEAs depends strongly on maintaining a refined and continuous FCC/BCC lamellar morphology. Unstable heat flow or improper energy input can transform the desired nanoscale lamellar structure into coarse cellular grains or discontinuous rod-like eutectic features. These unstable morphologies can intensify the local stress concentration, restrict coordinated deformation between the soft and hard phases, and promote premature cleavage fracture or reduced tensile ductility. A further severe limitation arises under notched or high-triaxiality loading conditions. Although EHEAs often show good strength and ductility under uniaxial tensile loading, the deformation incompatibility between the soft FCC phase and the hard BCC/B2 phase becomes more pronounced under triaxial stress. The resulting strain partitioning and local stress concentration at FCC/BCC interfaces can induce interfacial decohesion and microcrack nucleation. Once initiated, these microcracks may propagate rapidly along lamellar phase boundaries, leading to relatively low fracture toughness compared with that expected from their uniaxial tensile properties [107].

Compared with RHEAs, EHEAs, and precipitation-strengthened HEAs, single-phase FCC HEAs generally exhibit better processability and lower susceptibility to solidification cracking. Their major limitations are therefore less related to catastrophic printability failure and more associated with service performance. The first limitation is the strong defect sensitivity of fatigue life. Although FCC HEAs usually show high tensile ductility under monotonic loading, AM-induced defects such as gas pores, LoF defects, and near-surface pores can act as preferential fatigue-crack initiation sites. Even small near-surface defects can substantially reduce the high-cycle fatigue limit and lead to large scatter in fatigue data [80]. Another important limitation is the intrinsic strength–ductility trade-off of single-phase FCC HEAs. These alloys generally possess high plastic deformability, but relatively low initial yield strength compared with BCC, dual-phase, or precipitation-strengthened HEAs. Laser-based AM can partly overcome this limitation by introducing cellular dislocation structures, solute segregation at cell walls, refined grains, and high dislocation densities, all of which provide effective strengthening in the as-built state. However, these strengthening features are thermally unstable under high-temperature service or inappropriate heat treatment. Once extensive recovery or full recrystallization occurs, the AM-induced cellular structures, melt-pool boundaries, and dislocation networks can be largely eliminated. This microstructural degradation may cause a sharp decrease in yield strength and hardness, even though tensile ductility may improve [22].

In addition to alloy-class-specific limitations, reproducibility remains a cross-cutting challenge for all laser-based AM HEAs. Reproducibility is not controlled only by nominal laser parameters, such as laser power and scanning speed, but also by machine configurations, feedstock conditions, shielding atmospheres, build layouts, and accumulated thermal history. Even when identical process parameters are used, machine-to-machine variations can change melt-pool behavior because of the differences in laser beam calibration, spot-size stability, chamber gas-flow design, and spatter removal efficiency. These differences can alter local heat transfer, defect formation, and microstructural evolution, leading to scatter in density, crack density, tensile properties, and fatigue performance [80]. Build-platform placement and component geometry further limit data transferability. The position of a specimen on the substrate, its distance from the gas inlet and outlet, and the local heat-sink condition can modify thermal gradients and cooling rates. Therefore, small laboratory coupons and large engineering components may experience different thermal histories, even under the same nominal process window. This loss of similitude makes it difficult to directly use coupon-level mechanical data for component-level life prediction. For crack-sensitive alloys, especially RHEAs and precipitation-strengthened HEAs, the measured crack density can also vary with observation location, sectioning direction, and sample geometry. Standardized reporting of build orientation, platform position, inspected region, and component geometry is therefore necessary when evaluating printability and cracking susceptibility [80]. Feedstock variability is another important source of batch-to-batch scatter. The particle-size distribution of the powder must be carefully controlled because it affects powder spreading, packing density, laser absorption, and melt-pool stability. Excessive coarse particles may remain partially unmelted under a fixed energy density, especially in RHEAs containing high-melting-point elements. These particles can then act as stress concentrators and promote early fracture [52]. Powder reuse further complicates this issue. After repeated printing cycles, powders may undergo surface oxidation, morphology changes, satellite-particle formation, and mechanical damage during recoating and sieving. These changes can modify powder flowability, oxygen content, laser absorptivity, and defect formation, resulting in inconsistent densification and fatigue life among different powder batches. Shielding atmosphere and oxidation control are also critical for reproducible HEA fabrication. Different shielding gases and gas-flow conditions can affect heat transfer, plume behavior, spatter transport, and pore formation. Although Ar and N_2_ are commonly used, variations in gas purity, flow rate, and chamber design can still change melt-pool stability. Trace oxygen cannot be completely eliminated because it may originate from the chamber atmosphere, powder surfaces, or hollow powder particles. Even small oxygen variations can modify melt-pool surface tension and Marangoni convection, promoting balling, unstable melt tracks, and pore formation [31,80]. Therefore, maintaining a low and stable oxygen level, commonly below 100 ppm, is essential for suppressing oxidation-induced variability. Finally, thermal-history accumulation during layer-by-layer deposition creates additional uncertainty. Each deposited layer experiences repeated heating and cooling from subsequent laser passes, which can generate residual stresses and act as an in situ heat treatment. The extent of thermal accumulation depends, on part, the height, hatch strategy, interlayer time, substrate temperature, and component geometry [80].

Overall, the most critical limitations of laser-based AM HEAs are not universal but depend on alloy chemistry, phase constitution, and process reproducibility. RHEAs are mainly limited by incomplete melting, oxygen-induced grain-boundary embrittlement, and poor oxidation resistance. Precipitation-strengthened HEAs are primarily constrained by solidification cracking, liquation cracking, strain-age cracking, and the loss of volatile precipitation-forming elements. EHEAs are governed by a narrow VED processing window, selective evaporation of phase-stabilizing elements, and phase-morphology instability. FCC HEAs are comparatively printable, but their fatigue resistance and mechanical isotropy remain strongly affected by AM-induced defects, columnar grains, and melt-pool boundaries. Across all alloy classes, reproducibility further depends on powder reuse, particle-size distribution, oxygen control, shielding atmosphere, build-platform placement, component geometry, machine configuration, and thermal-history accumulation. These limitations indicate that future development should not rely on a single generalized process window. Instead, alloy-specific strategies integrating composition design, powder quality control, oxygen management, thermal-stress reduction, defect mitigation, vaporization control, standardized build reporting, and post-processing optimization are required to improve the reliability of laser-based AM HEAs.

### 8.2. Research Opportunities

Artificial intelligence (AI) and machine learning (ML) have emerged as powerful tools in designing HEAs and optimizing AM process parameters [93]. By leveraging data-driven approaches, critical characteristics such as densification, surface roughness, and mechanical properties can be precisely predicted and coupled with the optimization of laser parameters. Data-driven models have been applied to predict densification behavior, surface roughness, phase formation, hardness, tensile properties, and fatigue performance in laser-based AM alloys. In EHEA systems, ML models have also been used to forecast phase formation, hardness, and tensile behavior. To reduce the black-box nature of conventional ML, explainable AI methods, such as SHAP, have been integrated with thermomechanical data to evaluate the relative contribution of alloying elements, processing variables, and thermomechanical parameters to final properties [143]. These approaches provide a useful route for connecting data-driven prediction with physical metallurgy. Despite these advances, current ML frameworks for HEAs still focus mainly on composition–property relationships, while the effects of AM processing history are often simplified or neglected. This limitation restricts their practical use because the properties of laser-based AM HEAs are strongly governed by thermal history, melt-pool dynamics, defect formation, phase evolution, deformation mechanisms, and post-processing routes [144]. Therefore, predictive process–structure–property (PSP) modeling of laser-based AM HEAs should not rely on composition-based data alone. Instead, it requires a framework that links processing parameters, local thermal histories, microstructure evolution, defect formation, and mechanical responses. Physics-informed machine learning (PIML) provides one possible route to address this issue by embedding physical laws into data-driven models [145,146]. By incorporating governing equations, such as heat transfer, melt-pool fluid flow, and thermomechanical equilibrium, into the learning process, PIML can improve the prediction of transient temperature fields, residual stress evolution, and process-dependent material responses. Pre-trained PIML models may also reduce the computational cost of predicting new process conditions and can potentially act as soft sensors for process monitoring [146]. However, their transferability should be interpreted carefully because validation across different alloy systems, machine platforms, sensor configurations, and thermal histories remains necessary. In situ monitoring is another important direction for improving PSP prediction. Multimodal monitoring methods, including thermal imaging, acoustic emission, melt-pool monitoring, and X-ray-based inspection, can provide real-time information on thermal distribution, melt-pool stability, spatter behavior, pore formation, and defect evolution [147]. When combined with post-build microstructural characterization and mechanical testing, these data can support closed-loop process control and digital twin frameworks [148]. Nevertheless, the effective use of in situ monitoring requires standardized sensor calibration, synchronized data formats, and reliable links between sensor signals, microstructural features, and final properties. Integrated computational materials engineering (ICME) provides a physics-based framework for connecting thermodynamics, kinetics, process simulations, microstructure evolution, and mechanical responses. For example, CALPHAD-based calculations have been used to design the lightweight Ti_40_Zr_40_Mo_10_Al_10_ RHEA with a stable single-phase BCC structure over a wide temperature range from 860 to 1660 °C. When combined with finite-element thermal modeling, local thermal gradients and solidification rates in the melt pool can be extracted, helping to explain equiaxed grain formation under rapid cooling and gas-pore formation induced by Al vaporization [149]. By integrating thermal modeling, grain-growth modeling, diffusion and creep analyses, process optimization, and micromechanical modeling, ICME frameworks can predict thermal history, material states, defect formation, surface roughness, low-angle grain-boundary evolution, and mechanical properties [150]. Crystal plasticity finite element methods can further connect simulated microstructures with macroscopic mechanical responses [151].

However, several key knowledge gaps still limit reliable PSP modeling of laser-based AM HEAs. The first major gap lies in the process-to-structure linkage. During LPBF and LDED, microstructure formation is governed by highly transient and localized thermal conditions, including rapid melting and solidification, steep thermal gradients, melt-pool convection, recoil pressure, Marangoni flow, repeated remelting, and cyclic reheating. These factors control grain morphology, crystallographic texture, elemental segregation, phase morphology, precipitate evolution, defect formation, and residual stress. However, current process models often simplify one or more of these coupled phenomena [144]. For example, finite-element thermal models can estimate temperature fields and cooling rates, but they usually do not fully capture melt-pool fluid flow, vaporization, spatter formation, or pore entrapment. Similarly, cellular automaton and phase-field models can describe grain growth or phase evolution, but their coupling with realistic melt-pool dynamics, powder-bed behavior, and repeated thermal cycling remains limited. As a result, the accumulated effects of layer-by-layer thermal history on local microstructure, residual stress, liquation cracking, and precipitate coarsening are still difficult to predict quantitatively [152].

The second gap concerns the structure-to-property linkage. Even when the as-built microstructure can be characterized or simulated, translating it into reliable mechanical properties remains challenging. Laser-based AM HEAs often contain hierarchical and spatially heterogeneous features, including columnar grains, melt-pool boundaries, cellular dislocation structures, segregation networks, nanoscale precipitates, pores, LoF defects, and phase interfaces. Many of these features are three-dimensional and direction-dependent, whereas conventional characterization methods such as SEM and EBSD often provide only two-dimensional information. This mismatch makes it difficult to establish quantitative microstructural descriptors that can be directly used in mechanical models or data-driven frameworks [144,151]. In addition, current crystal-plasticity, mean-field, and continuum damage models still have limited ability to incorporate HEA-specific deformation and failure mechanisms, such as TWIP/TRIP interactions, deformation-twin thickening, dynamic dislocation pinning, slip transfer across phase boundaries, FCC/BCC interfacial decohesion, and pore-induced stress concentration. Therefore, predictions of ultimate strength, fracture toughness, fatigue crack initiation, and fatigue life remain less reliable than predictions of basic tensile properties [152].

The third gap is the lack of standardized data infrastructure for AM-HEAs. Reliable PSP modeling requires consistent process–structure–property datasets, but available AM-HEA data remain scarce, fragmented, and strongly dependent on individual laboratories or machines, which severely hinders the transferability of ML models across different AM platforms. Generating labeled AM data is expensive because it requires time-consuming experiments or high-fidelity simulations. In addition, compared with conventional alloys, HEAs are still a relatively young materials class and lack large, standardized, and open AM-specific datasets. Many reports do not consistently provide essential metadata, such as the powder particle-size distribution, powder reuse history, shielding gas purity, oxygen content, substrate material, preheating condition, scan strategy, build-platform position, heat-treatment history, and testing orientation. Consequently, alloys with the same nominal composition may show different microstructures, defect densities, or yield strengths because of unrecorded processing variations [148]. Current datasets are also biased toward successfully printed, near-equiatomic, single-phase FCC alloys, such as Cantor-type systems, whereas failed builds, crack-sensitive compositions, RHEAs, precipitation-strengthened HEAs, EHEAs, intermetallic-containing alloys, and amorphous structures are underrepresented. This publication bias limits not only ML training, but also the construction of reliable process maps, CALPHAD/kinetic validation datasets, fatigue databases, and qualification criteria [144].

The fourth gap concerns algorithm architecture and uncertainty quantification. Although emerging methods such as physics-informed neural networks (PINNs) offer a promising route for embedding physical laws into data-driven models, their practical application to laser-based AM remains technically challenging. High-fidelity physics-based simulations, such as coupled computational fluid dynamics–cellular automaton models, can capture melt-pool flow, solidification, and grain evolution, but their high computational cost makes them difficult to use for rapid process optimization or digital twin implementation [145]. In contrast, conventional ML models are computationally efficient but often lack physical constraints. PINNs attempt to bridge this gap by incorporating governing equations into the training process; however, under the highly nonlinear, high-dimensional, and multiscale conditions of laser-based AM, it remains difficult to simultaneously satisfy all relevant partial differential equations related to heat transfer, fluid flow, solidification, and thermomechanical responses. In addition, PINNs may suffer from spectral bias, where low-frequency features are learned more readily than high-frequency features. This can reduce their accuracy in capturing rapid melt-pool fluctuations, local fluid-flow instabilities, and microscale thermal variations [145]. Another unresolved issue is the lack of physical interpretability and uncertainty quantification. Many current AI-based AM models still provide deterministic point predictions without confidence intervals or reliability bounds. Therefore, uncertainty quantification approaches, such as Bayesian inference, Gaussian processes, and ensemble learning, are needed to identify unreliable extrapolation and improve the applicability of predictive models in safety-critical fields such as aerospace and biomedical manufacturing [93,144].

Therefore, future development should integrate AI/ML, PIML, in situ monitoring, and ICME within a unified PSP framework. ICME can generate physically consistent synthetic data, including thermal histories, three-dimensional microstructures, defect distributions, and stress–strain responses, thereby helping to reduce data scarcity. Conversely, ML can serve as a fast surrogate model for computationally intensive ICME simulations, enabling rapid screening of compositions and process windows [124,130,131]. In situ monitoring can provide real-time process information for defect detection and closed-loop control, while PIML can incorporate physical constraints into data-driven prediction. However, these approaches require open and balanced AM-HEA datasets, standardized metadata protocols, validated three-dimensional microstructure descriptors, defect-aware mechanical models, experimentally validated synthetic data, and uncertainty-aware prediction frameworks. Such an integrated physics–data framework is essential for moving from empirical process optimization toward reliable predictive modeling of laser-based AM HEAs. 

### 8.3. Industrialization Barriers

Although laser-based additive manufacturing shows strong potential for fabricating complex-geometry HEA components and tailoring their microstructures, translating this technology from laboratory-scale studies to industrial production still faces several practical barriers. Manufacturing costs remain an important concern, especially for cost-sensitive sectors such as automotive, electronics, and construction. Compared with mature casting, forging, and wrought processing routes, laser-based AM requires expensive equipment, premium spherical multi-element powders, high energy consumption, inert gas protection, labor-intensive post-processing, and relatively low build efficiency. These factors make the overall production cost of AM-processed HEAs substantially higher than that of conventional manufacturing routes [54,74]. Second, ensuring process reproducibility represents a major technical hurdle. Laser-based AM processes involve extreme thermal gradients, rapid thermal cycles, and complex laser-feedstock interactions, which inevitably lead to batch-to-batch variances during fabrication [31]. The high sensitivity of melt-pool dynamics to processing parameters often triggers the stochastic formation of undesirable metallurgical defects (e.g., porosity and LoF voids) [80]. Consequently, this lack of reproducibility and the stochastic nature of internal defects create immense challenges for parts’ qualification and certification. In safety-critical applications, the stringent certification processes currently rely heavily on expensive, time-consuming destructive testing and trial-and-error approaches. Until robust uncertainty quantification and standardized defect-control frameworks are established, obtaining industrial certification for AM-fabricated HEA components remains a formidable obstacle. Beyond the cost, qualification standards and certification remain major obstacles. Current ASTM and ISO standards for metal AM are still developing, and alloy-specific standards for HEAs are largely unavailable [31]. Standardized protocols are still needed for powder characteristics, powder reuse, process parameters, shielding atmosphere, heat treatment, post-processing, defect acceptance criteria, and mechanical testing. In addition, the transferability of laboratory data to industrial components is limited by the loss of similitude. Small test coupons and large engineering parts usually experience different heat dissipation conditions, thermal histories, residual stress states, and microstructures. Therefore, mechanical properties measured from simple specimens cannot always be directly used for life prediction of complex components. This issue is particularly important for safety-critical applications, where certification requires reliable links among processing history, defect population, microstructure, and long-term performance [80].

Nondestructive evaluation and in situ monitoring are also critical for industrial qualification. Laser-based AM parts often contain randomly distributed internal defects, such as LoF voids, gas pores, keyhole pores, and near-surface defects, which can serve as crack initiation sites. However, conventional nondestructive evaluation methods, including ultrasonic inspection and X-ray radiography, have finite detection limits. These limits directly affect the effective initial flaw size used in damage-tolerant design. High-resolution X-ray computed tomography can provide three-dimensional defect information, but its high cost and low throughput restrict its use mainly to research or high-value components [80]. Surface finish and dimensional tolerance also present additional barriers. Because of powder adhesion, balling, partially melted particles, and unstable melt-pool flow, LPBF and LDED components often exhibit rough surfaces. These surface irregularities act as local stress concentrators and can strongly reduce high-cycle fatigue life [80]. Moreover, steep thermal gradients and rapid cooling generate high residual stresses, which may cause distortion during fabrication, substrate removal, post-processing, or service. Such distortion makes it difficult to meet strict dimensional tolerance requirements for precision components [31]. As a result, additional machining, polishing, or surface-strengthening treatments, such as laser shock peening, ultrasonic nanocrystal surface modification, or related surface treatments, are often required. These steps improve surface integrity and dimensional stability but further increase manufacturing costs and process complexity.

Repairability is another important industrial consideration. LDED is attractive for repairing high-value components, such as turbine blades or locally damaged structural parts. However, reliable repair requires strong metallurgical bonding between the deposited material and the substrate, controlled dilution, and stable microstructure across the repaired region [72]. The repaired zone may contain epitaxially grown grains, orientation mismatch, heat-affected zones, and local segregation. Therefore, the long-term fatigue, creep, and environmental durability of repaired HEA components remain difficult to predict. Environmental exposure and service stability must also be considered before industrial deployment. In practical service, AM components may be exposed to high temperature, water vapor, oxidizing atmospheres, corrosive media, or coupled mechanical and chemical loading. Surface-connected defects and microcracks can accelerate localized corrosion and stress corrosion cracking [99]. Therefore, environmental degradation, oxidation behavior, corrosion resistance, and stress corrosion cracking should be evaluated together with tensile, fatigue, creep, and fracture properties.

Scale-up remains a further limitation. LPBF provides high precision but relatively low deposition rates, which limits its use for large-volume production [54]. Multi-laser systems can increase productivity, but they also introduce additional challenges, including laser-to-laser calibration, overlapping thermal fields, spatter interaction, and process stability. In contrast, LDED and wire-based AM can fabricate larger components with higher deposition rates, but their lower dimensional accuracy and rougher surface finish often require extensive subtractive machining [80]. The build-chamber size, powder-bed volume, recoating stability, and parts’ geometry also restrict the fabrication of large integrated structures. Finally, life-cycle assessment and sustainability should be included in future industrial evaluations. Although AM can reduce material waste for complex parts, its specific energy consumption can be high. From a cradle-to-gate perspective, metal powder production, high-purity shielding gas consumption, HIP, heat treatment, machining, and powder recycling all contribute to a cumulative energy demand and environmental impact. Powder reuse can improve material efficiency, but repeated thermal exposure and handling may cause oxidation, morphology degradation, and changes in flowability, which introduce uncertainty in both parts’ quality and environmental benefit [74]. Therefore, future industrialization of laser-based AM HEAs should be evaluated not only by mechanical performance and cost, but also by qualification standards, nondestructive evaluation capability, repair reliability, surface integrity, dimensional accuracy, environmental durability, scalability, and life-cycle impact.

### 8.4. Prioritized Research Directions

Based on the above challenges, future research on laser-based AM HEAs should be prioritized in several directions. First, standardized and open AM-HEA databases should be established as the foundation for predictive process–structure–property modeling [144]. These databases should include not only composition and mechanical properties, but also the powder characteristics, reuse history, oxygen level, shielding atmosphere, build layout, thermal history, post-processing conditions, defect statistics, and three-dimensional microstructural descriptors. Such data infrastructure is essential for reducing publication bias and improving the reliability of AI/ML, CALPHAD, ICME, and uncertainty-aware models.

Second, alloy design should shift from defect elimination alone toward defect-tolerant strategies. Since completely removing pores, LoF defects, and microcracks is difficult during industrial AM production, future HEAs should be designed to resist crack initiation and growth. Metastable FCC HEAs that activate TRIP or TWIP near defect tips are promising candidates, as local phase transformation or deformation twinning can relax stress concentration, increase work hardening, and improve fatigue resistance [124].

Third, oxygen-resistant and crack-free RHEAs should be developed for high-temperature applications. Although RHEAs provide high-temperature strength, they are highly sensitive to oxygen-induced grain-boundary embrittlement during AM. Future composition designs should focus on grain-boundary engineering, such as controlled B or C microalloying to improve boundary cohesion and suppress oxygen segregation [102]. Another promising route is to understand and control ordered oxygen complexes, which may transform harmful interstitial oxygen into a strengthening contribution rather than a source of embrittlement [153].

Fourth, in situ monitoring and closed-loop control should be advanced from defect detection to real-time process correction [154,155]. Multimodal sensing, including high-speed thermal imaging, optical monitoring, acoustic emission, and X-ray-based diagnostics, should be integrated with ML and digital twin models. A key target is the development of low-latency control systems that can identify keyhole instability, spatter formation, overheating, or LoF conditions and dynamically adjust the laser power, scan speed, or hatch strategy during fabrication.

Fifth, post-processing strategies should be designed to retain beneficial AM-induced microstructures. Cellular dislocation structures, nanoscale segregation networks, and fine precipitates are important strengthening features in many laser-based AM HEAs. Conventional high-temperature HIP or annealing can heal pores and reduce residual stress but may also remove these strengthening structures and cause a sharp decrease in yield strength. Future post-processing should therefore decouple defect healing and stress relief from thermal coarsening, for example, through short-time controlled annealing, optimized HIP cycles, laser shock peening, or combined surface mechanical treatments. These prioritized directions can support the transition of laser-based AM HEAs from empirical process optimization toward reliable, certifiable, and application-specific materials design.

Overall, the future development of laser-based AM HEAs requires a coordinated framework that connects CALPHAD-based thermodynamic prediction, ICME-based process–structure–property modeling, AI/ML-assisted data analysis, and in situ monitoring with closed-loop process control. Rather than relying on generalized processing parameters, alloy-specific and defect-control-oriented process windows should be established using standardized AM-HEA databases, reproducible metadata reporting, and validated qualification protocols. In parallel, post-processing strategies should be designed not only to reduce defects and residual stresses but also to retain beneficial AM-induced microstructures, such as cellular dislocation structures, nanoscale segregation networks, and fine precipitates, where they contribute to mechanical performance. Such integration will be essential for translating laser-based AM HEAs from laboratory demonstrations to reliable industrial components with predictable mechanical performance, environmental stability, and service life.

## Data Availability

No new data were created or analyzed in this study. Data sharing is not applicable to this article.
